# Conversations Surrounding the Use of DNA Tests in the Family Reunification of Migrants Separated at the United States-Mexico Border in 2018

**DOI:** 10.3389/fgene.2019.01232

**Published:** 2019-12-13

**Authors:** Jennifer K. Wagner, Diana Madden, Valedie Oray, Sara H. Katsanis

**Affiliations:** ^1^Center for Translational Bioethics & Health Care Policy, Geisinger, Danville, PA, United States; ^2^Mary Ann & J Milburn Smith Child Health Research, Outreach, and Advocacy Center, Ann & Robert H Lurie Children’s Hospital of Chicago, Chicago, IL, United States; ^3^Initiative for Science & Society, Duke University, Durham, NC, United States; ^4^Department of Pediatrics, Feinberg School of Medicine, Northwestern University, Chicago, IL, United States

**Keywords:** public understanding of science, science communication, social media, kinship analysis, DNA testing, forensic DNA, immigration, border policy

## Abstract

In April 2018, the U.S. implemented a “zero-tolerance” immigration policy that would lead to the separation of more than 2,000 migrant families over the following months. By that summer, the policy and resultant family separations had generated a media storm that swept up the public. In early June, the government announced its consideration of DNA testing to aid in the detection of human trafficking in immigration contexts. Later that month, as the government retracted the child separation policy, the public began questioning how children and adults would be reunited and discussing the potential usefulness of DNA testing for those reunifications. Then in early July, the government announced that DNA testing was indeed being used, and by mid-month the public’s outrage over the use of DNA was strong. We set out to examine the public dialogue on DNA testing—including misunderstandings and miscommunications—both in newspaper coverage and on Twitter in the 2-month summer period of 2018, at the height of public discussion of migrant family separations and then reunifications. We performed database searches identifying 263 newspaper articles and used Twitter’s advanced search function identifying 153 Tweets containing discussion of the use of DNA for migrant family reunification. Upon the resulting sources, we performed content analysis, analyzing for slant on the immigration policy and the use of DNA tests using a combination of open and closed codes. Our analysis showed that perspectives on the use of DNA diverged in connection with perspectives on the immigration policy, and that there was a contrast among the cohorts in the stated utility of DNA testing. These findings offer insight into a) how DNA testing in a highly politicized immigration context was represented in media coverage and b) the public’s understanding of the role that DNA testing could or should play in immigration. By detailing the role that comments from experts, stakeholders, and the public played in these discussions, we hope to provide lessons for communications with the public about future non-medical applications of genetic technologies.

## Introduction

The first DNA test using PCR of short tandem repeats (STRs) was for a case published by Alec Jeffreys in 1984 to prove parentage between a Ghanaian child and his parents for immigration purposes (Jeffreys et al., 1985). Since the 1980s, most Western countries have expanded the use of DNA testing in immigration contexts (Weiss, 2011). Over 30 years later, in the summer of 2018, the DNA testing of families at the United States-Mexico border became one of the focal points of an international public debate over the “zero-tolerance” policy that criminalizes crossing the border and resulted in the separation of children from adult family members. Journalists and reporters from a wide range of media covered the story, contacting scientists and experts for input—including two of us authors (Molteni, 2018; Ray, 2018). The apparent misconceptions in the media prompted us to pen an opinion editorial just prior to the declared retraction of the family separation policy (Katsanis and Wagner, 2018). Interested in the role of DNA testing as perceived in the public sphere, we were motivated to perform an autopsy of the cascade of events in June and July 2018 (Farahany et al., 2019). Because use of DNA testing in immigration is not new, we wanted to understand how the public was talking about the use of genetic information during this timeframe and examine the accuracy of the public dialogue in the news and social media.

For years the United States immigration system has permitted the voluntary use of DNA tests as evidence for family relationships that might lack substantial paper or electronic documentation (USCIS, 2000). In 2008 the federal government expanded the collection of DNA for the federal criminal database (CODIS, Combined DNA Index System, FBI, Quantico, VA) to include persons detained upon entry to the United States (73 Federal Register 74932, 2008). Refugees and asylum seekers petitioning under the Priority-3 (P-3) segment of the U.S. Refugee Admissions Program have been required for more than 5 years (75 Federal Register 54690, 2010) to provide a DNA test to verify claimed familial relationships, when the DS-7656 Affidavit of Relationship was modified before the P-3 program was relaunched following a 4-year hiatus (78 Federal Register 70313, 2013). This model was applied to the now-defunct (82 Federal Register 38926, 2017) Central American Minors Refugee/Parole (CAM) program (79 Federal Register 68343, 2014), requiring DNA testing to verify parentage of children in El Salvador, Guatemala, and Honduras seeking to join their parents in the United States (Bureau of Population, Refugees and Migration, 2014; U.S. Citizenship and Immigration Services, U.S. Department of Homeland Security, 2017).

The zero-tolerance policy announced by the U.S. government on April 6, 2018 put in place procedures to detain and prosecute all migrants that crossed the southwest United States border illegally (U.S. Department of Justice, 2018). Justifications for the policy included the purported need “to restore legality to the system,” (Sessions, 2018a) to treat everyone who crosses without authorization at an official port of entry the same—without exception (Blitzer, 2018), and ultimately to deter future illegal border crossers (Hirschfield Davis and Shear, 2018; Sessions, 2018a; Congressional Research Service, 2019). Alongside the policy was a motion to separate children from the adults who brought them over the border, including parents and other relatives. There is no evidence that this policy requiring separation of children from their accompanying adults was applied to the northern border with Canada or to other ports of entry (such as airports) (Sessions, 2018b). The separation of children from accompanying adults continued as a part of the order until June 20, 2018, when President Donald J. Trump signed an executive order declaring an end to the practice (Trump, 2018).

Shortly following the executive order, companies publicly offered DNA testing services, equipment, and reagents to help with family reunification (Brandom and Becker, 2018). For example, 23andMe and MyHeritage both offered use of their personal genome services (Staff, 2018a; Brandom and Becker, 2018; Peters, 2018). Thermo Fisher Scientific offered access to their equipment and/or reagents (Staff, 2018b). To our knowledge and by all public accounts, neither the government nor non-governmental organizations (NGOs) accepted these offers. Nevertheless, on July 5, 2018, U.S. Department of Health and Human Services (HHS) Secretary Alex Azar announced in a conference call with the press that the Office of Refugee Resettlement (a branch of HHS) would be using DNA tests to reunify families who had been separated at the border (Brown, 2018; Flores, 2018).

The family separation crisis that began in 2018 is ongoing, with many children still separated from their families (Lind, 2019), new families reportedly being separated (Hoffman, 2019; Jordan and Dickerson, 2019), several claims of abuse of children whilst in custody (Gonzales, 2019; Haag, 2019), and even reports of separated children being placed for adoptions (Raff, 2018). The Trump administration appears to be holding fast to its desire to detain and deport unaccompanied children more easily (Lanard, 2017) by seeking additional authority from Congress to lengthen allowable detention periods, make it easier to deport migrant unaccompanied children from countries not sharing a border with the United States, and require asylum seekers to apply for protection from within their home country (Feuerherd, 2019; Madan, 2019). The consequences of the zero-tolerance policy that separated families will reverberate in the coming years, and it is important for geneticists to reflect upon the role of DNA testing in contributing to and/or alleviating the crisis.

News media and social media were key public fora in which these events were described and debated, shaping the public’s understanding of not only the zero-tolerance policy specifically but also the role of DNA testing in immigration generally. The news media is influential in determining which issues receive public attention and conveys opinions that members of the public might adopt on an issue (McCombs and Shaw, 1972). Most adults use news media as their main source of information; this is especially true for information in the scientific realm, where Americans tend to rely on news coverage of important scientific issues and discoveries (National Science Board, 2016). However, a great proportion of Americans lack scientific literacy (Maienschein, 1999), making it a challenge for many to synthesize scientific information and leading to a disconnect between the scientific community and the general public (Weigold, 2001). An analysis of media coverage can provide scientists and administrators insight into how science is understood by the journalists and reporters who cover their areas of interest and then communicated to the broader public (Geary et al., 2016).

Social media also has come to play an important and increasingly acknowledged role in news consumption and public policy discussion; two-thirds of American adults report that they follow news on social media at least occasionally (Matsa and Shearer, 2018). Twitter, Inc. (San Francisco, CA), as one of the largest social networks, provides about 12% of Americans with news, and 71% of its users read news on the platform (Matsa and Shearer, 2018). While news inaccuracy on social media platforms is a common concern (Mitchell and Barthel, 2017), most users still see advantages to consuming news through this medium (Matsa and Shearer, 2018). Not only do journalists, news outlets, organizations, and private users share and discuss news items on Twitter, it is also the most popular social media platform among both United Nation member states (Lüfkens, 2018) and government agencies within the United States. President Donald J. Trump in particular has harnessed the platform to broadcast new policies as well as his stances on government matters, which in turn has given credence to Twitter as an appropriate communication tool for public policy dialogue. Twitter—with its immediacy, interactivity, and diverse plurality of voices—is a rich complement to news articles and provides further insight into the generation of, consumption of, and reception of science and science policy-related news.

Following the summer 2018 international conversations on the use of DNA in this discrete non-medical context, we were interested in observing how the public discussion played out, specifically with regard to the use of DNA testing for family reunification in the U.S. immigration system. We sought to capture the news media and a sampling of Twitter conversations between June 1 and July 31 of 2018 and to evaluate biases in the dialogue, the scope of the conversations around the DNA testing processes, and the accuracy of the information being conveyed. Our initial aim was to capture a snapshot of 1) what the scientific community was communicating to the news media about DNA testing and 2) how the public was responding to this information through social media.

## Materials and Methods

### News Articles and Tweets as Complementary Source Material

**Figure 1:**
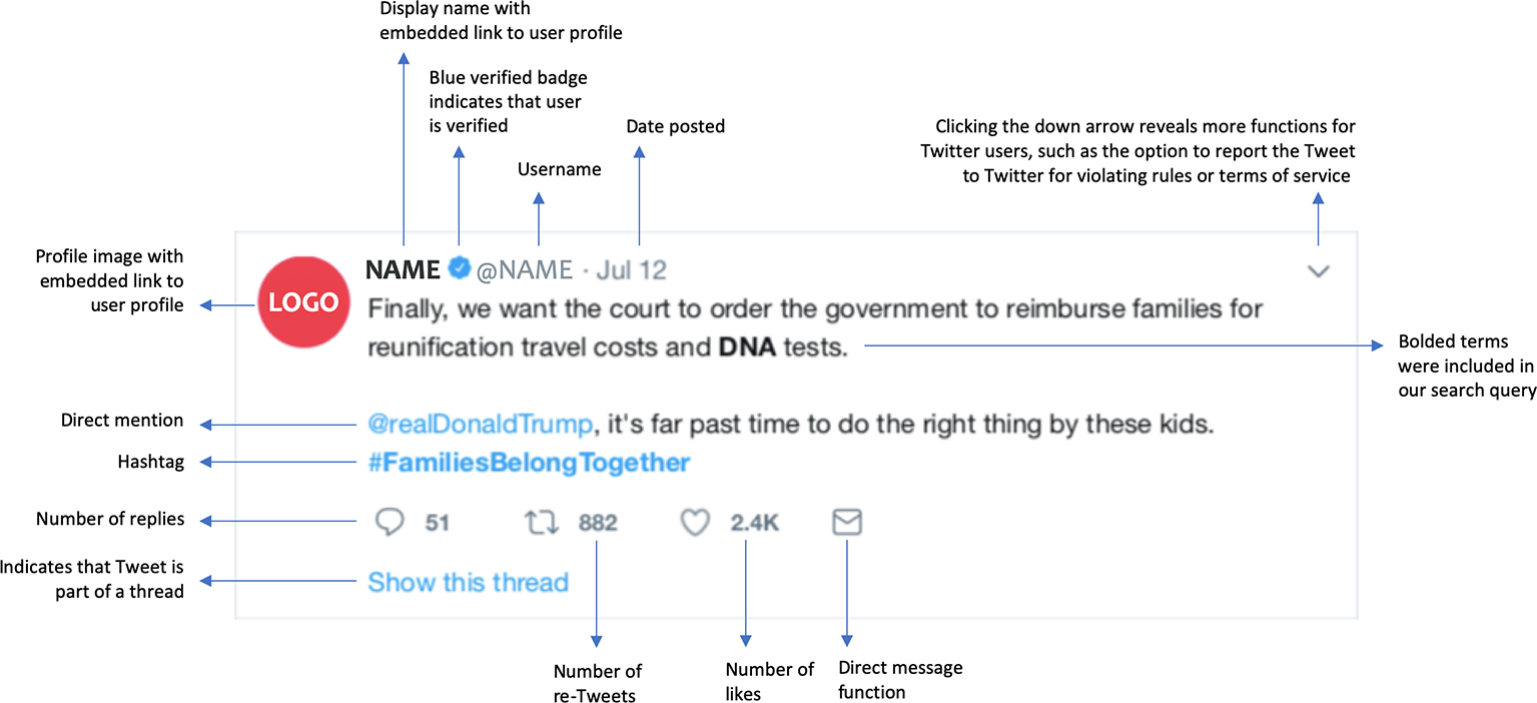
Anatomy of a Tweet. A Tweet as it appears in a Twitter feed, timeline, and search results. A display name identifies a user to other users, while a username is the unique ID associated with an account; these names might differ. The blue verified badge is applied by Twitter to indicate that accounts of public figures and organizations are authentic. The direct message function allows Twitter users to send each other private messages. The re-Tweet function allows users to re-post their own or another user’s Tweets, sometimes with additional commentary, while a reply is a direct response to a Tweet. In addition to being a reply or a re-Tweet, a Tweet might also be part of a thread, as indicated here by “Show this thread.” A thread is created when a user connects a series of their Tweets. Hashtags—a word or phrase marked by the # symbol—are often key words or topics in a Tweet that can be used to locate Tweets from different users covering the same topic. Direct mentions, marked by the @ sign, allow users to direct their published Tweets at particular Twitter users or call out particular users. All descriptions and definitions here are drawn from our exploration of Twitter and https://help.twitter.com/.

Analysis of data gathered from the social media platform Twitter was conducted parallel to analysis of newspaper articles. While the same methodology was applied to both research venues, differences in accessibility and format of materials required different approaches to data collection and analysis. The contrasts and connections between our chosen venues allowed us to capture a broad snapshot of public dialogue around the zero-tolerance policy and DNA testing in summer 2018. We opted to examine news articles, as long-form pieces with journalistic authority, to capture static dialogue from a traditional venue. Twitter, on the other hand, is a newer form of media. With its self-published, short-form missives (Tweets are limited to 280 characters), Twitter is both a popular social media platform and an increasingly acknowledged forum for public policy dialogue. Tweets thus provide a rich contrast to our captured news items (see Figure 1). The potential connections between the two venues—as journalists and news outlets use Twitter and as Twitter users read and share news articles—linked the overlapping analyses.

### News Analysis

#### Data Access and Collection

A search of ProQuest U.S. Major Dailies newspaper database (which includes the five newspapers: *The New York Times*, *Washington Post*, *The Wall Street Journal*, *Los Angeles Times*, and *Chicago Tribune*) using the search string DNA + migrant (appearing anywhere) was performed on August 8, 2018 by JW. Results were limited to those articles occurring between June 1 and July 31, 2018 and to those categorized by ProQuest as newspaper items (rather than blogs, podcasts, or websites). In efforts to expand the search results, a trio of systematic searches were performed by JW on August 10, 2018 using ProQuest, Westlaw Next (Thomson Reuters; querying “all U.S. newspapers”), and Nexis Uni (Lexis Nexis; querying the “U.S. news” option materials in English language) and relying upon the same search string and temporal restrictions as the earlier search of ProQuest. The newspaper items identified systematically were combined with an *ad hoc* set of items known to JW and SK at that time.

#### Preliminary Content Analysis

Relevant and unique news items were screened by three investigators (SK, DM, and JW) for scope of content to examine to what extent DNA testing was covered as a topic. Articles that simply mentioned that DNA testing was being used were screened out from further analysis, and a codebook was developed to aid in the screening of the news content (see **Supplemental File** “*Codebook for News*”, **Data Sheet 1**). The coders read for key words to indicate some coverage not just that DNA testing was being considered or used but whether DNA testing should be considered or used (for example, one article stated, “Azar said Thursday morning that an army of government workers had been dispatched to review files and conduct DNA testing to match parents with their children” (Sacchetti, 2018a). This text was insufficient to evaluate whether DNA testing would be useful, ethical, or legal, so this article was excluded. The same author in another article stated, “Sabraw had earlier ordered the government to forgo DNA testing of all adults and to skip background and fingerprint checks of all residents in the homes where the children will live, unless there was a safety concern” (Sacchetti, 2018b). This text was sufficient to be included.). Articles that had some context for DNA testing were further coded by two investigators (JW and SK) for slant of the article (pro- or anti-) with regard to immigration, zero-tolerance policy, and DNA testing. Key words were documented to substantiate the identified slant. These articles were further coded for what category of experts were consulted on the DNA testing aspect of the article and what DNA testing-related topics were covered. Finally, the articles were coded for whether they addressed any topics regarding the science of DNA testing, the legal authority for DNA testing, or the ethical considerations for DNA testing. These articles comprised the set of news items for in-depth content analysis.

#### In-Depth Content Analysis

We developed a set of codes that would be used for content analysis based on observing important topics of interest (see **Supplemental File** “*Codebook for News and Twitter Content*”, **Data Sheet 3**). These topics were broadly categorized into ethical issues, scientific processes, legality, and other considerations. Codes were devised in most cases on a yes-no basis as to presence of the topical content in the article. Other codes were more specific, such as the topic on method of DNA collection for which the specified types of collection mentioned in each article were recorded. Two coders (SK and VO) independently coded each article utilizing the codebook and resolved any discrepancies found between their analyses. Some codes underwent minor adjustments in order to provide clarity as comparisons were made between the two code sets during this reconciliation process. Content analyses were inserted into Microsoft Excel and tabulated based on the devised codes. A set of research questions and hypotheses were formulated based on the data to extrapolate news content trends.

#### Use of Bias Assessors

Given the potential bias of news sources (Iyengar and Massey, 2018; Pennycook and Rand, 2019), we sought to include third-party evaluation of the news sources to gauge bias. Because there is no standard news source bias checking resource (Pingree et al., 2018; Pennycook and Rand, 2019), we reviewed the methodologies and funding sources of several bias-checking resources and selected three of the most prevalent, on-going resources aimed at offering a comprehensive overview of media bias in North America. The metrics of each resource to evaluate the news sources were incorporated where possible into our data analysis (see **Supplemental File** “Media Bias Assessors”, **Data Sheet 4**). The sites selected were 1) AllSides (www.allsides.com); 2) Media Bias/Fact Check (mediabiasfactcheck.com); and 3) Ad Fontes Media, Inc. (www.adfontesmedia.com). We compared bias ratings across all three sources where possible.

### Twitter Analysis

#### Data Access and Collection

Both prospective and retrospective searches of Twitter present challenges, but barriers to access are higher for historical data (i.e., Tweets already published), which was what our study necessitated. A comprehensive dataset drawn from a search run against the full archive of Tweets for a timeframe of interest would be accessible only through a third-party Twitter service provider or through paid-access to the Twitter application programming interface (API); both of these services are cost-prohibitive and generally are designed for commercial market research, not social science research. More importantly for our research, Twitter’s free standard API searches result in outputs of an unspecified proportion of all Tweets published in the prior seven days. This approach was not useful for our purposes since we wanted to capture the discussion during a particular 2-month time period.

We opted to gather our data using Twitter’s “Advanced Search” user interface, which is freely accessible to anyone with a Twitter account. The Advanced Search user interface pulls Tweets for any time period or range of time; however, the search algorithm is proprietary so the details on how the search captures data is unclear. Like the standard search API, the user interface searches for relevance of the results to the search terms, not completeness (meaning some Tweets might be missing from search results) and Twitter does not detail how precisely results are generated (Twitter, 2019). Nevertheless, an Advanced Search that is specific enough will provide a snapshot of the Twitter dialogues for a particular time period around a particular topic; however, it is not necessarily identical to the Twitter traffic of that time period had it been collected in real time. This is due, for example, to the frequency of Twitter users changing their own profiles or deleting content, such that the results might be altered at a later date. For our purposes, we settled on carefully refining our Advanced Search parameters and performing two one-time searches to generate a sampling of Tweets indicative of the Twitter dialogues on our topic of interest.

Search queries were developed by running string searches for relevant key words. Once the refined searches demonstrated their capacity to provide relevant content, we compiled a list of frequently used hashtags from the results. The most common and topically relevant of these were selected for the final searches. Two final one-time searches of Twitter were conducted by SK on August 17, 2018 using the Advanced Search user interface: 1) a search of “DNA” plus any of 17 trending hashtags (#keepfamiliestogether; #familiesbelongtogether; #reunitefamilies; #reunitethefamilies; #reunitefamiliesnow; #reunification; #separationoffamilies; #childrenincages; #familyseparation; #reuniteeverychild; #returnthechildren; #bordercrisis; #childrensconcentrationcamps; #childtrafficking; #humantrafficking; #illegalimmigration; and #buildthewallnow) and 2) a search of “DNA” plus any of five terms relevant to migration (“illegals,” “immigrant,” “immigration,” “migrant,” and “refugee”). Parameters were set to search Twitter posts between June 1 and July 31, 2018; the language filter was set to English; and the quality filter (a feature that filters out what Twitter calls “lower quality content,” including duplicate Tweets or seemingly automated content) was turned on. Each search yielded a set of Tweets in chronological order, which we then saved as portable document formats (PDFs). This method of capturing search results successfully pulled all results simultaneously; preserved hyperlinks within Tweets as well as hyperlinks embedded in the Twitter users’ profiles, hashtags, direct mentions, threads, and re-Tweets; and preserved hyperlinks to each Tweet embedded in the date. One drawback was that the PDF archive failed to capture emojis and images consistently.

The text of each Tweet was subsequently entered manually into a Microsoft Excel spreadsheet. Tweets were coded for a set of closed codes focused on set characteristics of Twitter data (e.g., Twitter name, Twitter handle). Two coders (SK and DM) screened for duplicate Tweets and irrelevant Tweets to eliminate them on the basis of the text of the Tweets alone. Tweets that did not address the use of DNA testing in the context of immigration were considered irrelevant. Tweets from public figures or organizations were not excluded.

#### Content Analysis

##### Codebook

An initial codebook consisting of a mix of closed and open codes was developed on the basis of research interests in slant and use of DNA testing, the infrastructure of Twitter, and quantitative metrics for Twitter from the methodological literature (see **Supplemental File** “*Codebook for Twitter*”, **Data Sheet, 2**) (Murthy, 2016). Throughout the coding process, the codebook was adapted to accommodate shifts in data interest and to parse details not considered by investigators at the outset. Set characteristics of Tweets comprised our closed codes, with the only alterations to these codes being the removal or addition of categories. For qualifying users as individuals or as “bots” (automatic robotic Twitter users rather than human interfacing users), we made some efforts to screen each account to determine if it might be a bot. After extensive research on Twitter bots and how to determine if a user was a bot, we opted to depend on Twitter’s quality filter to weed out the majority of bots rather than systematically evaluating each user. Codes for Tweet content comprised our open codes, including codes for Tweet purpose and slant. All coding was completed by two independent coders (SK and DM) and then reconciled through comparison and discussion. After an initial round of coding, we turned to deeper content analysis of the Tweets by establishing total counts for the use of hashtags and directed mentions; listing the terms used to refer to migrants; listing the actors and organizations mentioned; and applying codes for concepts covered developed for news coverage to Tweets for comparison.

##### Rulebook

In addition to developing a Twitter codebook, we also developed a rulebook as a guide to the Twitter coding process. We borrowed the concept of a rulebook from Brubaker *et al*., who describe the process of developing “codebook rules” to aid them in determining whether to code MySpace comments as containing emotional distress (Brubaker et al., 2012). The Twitter rulebook contains both a guide to the coding process and rules designed to prevent biased coding (see **Supplemental File** “Codebook for Twitter”, **Data Sheet 2**). Several unique features of Twitter data necessitated the creation of the rulebook. Firstly, a Tweet is never purely textual data. The text of a Tweet is embedded in the infrastructure of Twitter (see **Figure 1** for a detailed explanation of the elements that Tweets contain). Every Tweet contains both non-textual data as well as hyperlinks to contextualizing data (e.g., Twitter user profile, news items, other Tweets). The guide to the coding process not only lays out the order in which codes should be addressed, it also sets the order in which the elements of a Tweet should be examined and how much weight should be given to each. The rules give further instruction on how to treat various Tweet elements. Furthermore, Tweets, with their short length, lack of immediately available textual context, and casual tone (Puschmann et al., 2013; Osmond, 2017) force researchers to confront ambiguities during the coding process. This is especially true when researchers are coding for complex and slippery concepts such as political bias or views on immigration issues (what we call slant, more below). The rulebook is a tool to prevent coders from unconsciously relying on their own biases to determine codes where evidence to support a code is ambiguous or scarce. It also served as a place for coders to concretize a consistent approach to Twitter data by documenting the coding strategies that emerged through discussion. The rulebook was first developed once the codebook was drafted and codes were given initial definitions. Following Brubaker *et al*.’s lead, we pulled Tweets with the most explicit expressions of views on politics, the zero-tolerance policy, and DNA testing. Their characteristics were described to generate the first draft of the rulebook, which was refined and expanded through code reconciliation and discussion throughout the process.

##### Tweet Interpretation

Twitter’s format requires innovative approaches to content analysis. Not only are Tweets short (currently limited to 280 characters), but also, they are often difficult to decipher due to their casual tone; misuse of punctuation; use of incomplete sentences, use of slang; use of acronyms and short-hand; and use of Twitter conventions, including hashtags, direct mentions, and emojis. The lack of textual data within individual Tweets is contrasted with a relative wealth of non-textual data in the form of images, emojis, memes, photographs, and hyperlinks, as well as quantitative metrics (e.g., the number of re-Tweets, likes, and replies for each Tweet). The infrastructure of Twitter also provides context: each Tweet in our dataset was linked to a profile and might itself be a re-Tweet, a reply, or part of a thread; a response to an ongoing discussion on Twitter or in the media at large; or a pointed comment directed at another Twitter user.

We drew on non-textual and contextualizing data to develop an interpretation of the text of each Tweet into full sentences that clarified and encapsulated the intention and message of the Tweet. This process allowed the researchers to review two items of text for analyses: 1) the original Tweet text for analysis of slant and use of certain words, hashtags, or direct mentions and 2) the interpreted Tweet text for analysis of intended content and purpose, viewed without the potentially divisive rhetoric. Two independent coders (SK and DM) reviewed each Tweet for set characteristics in order to familiarize themselves with the immediate context. If the Tweet contained images, was a reply, a re-Tweet, part of a thread, or hyperlinked to an article, the images and hyperlinked Tweets or content were examined. Finally, key words as well as slang terms, abbreviations, hashtags, or direct mentions requiring clarification were identified and punctuation was examined. The Tweet was then rendered into full sentences in neutral language, with each sentence beginning with verbs that indicated the purpose of the Tweet (e.g., comments, questions, or shares). The two sets of interpretations were then reconciled and any discrepancies discussed to ensure that both coders agreed on the final interpretation of the Tweet. While effective, this method was not foolproof. We were careful to note Tweets where multiple interpretations were possible. The list of verbs generated by the final interpretations was refined and became the list of “purpose” codes for Tweets.

#### Analysis of Slant

As in the news article analysis of slant, each Tweet was coded by two coders (SK and DM) for slant including pro-/anti-zero tolerance policy and pro-/anti-DNA testing. Additionally, Tweets were evaluated for political bias. Codes for slant and political bias were not conceptualized as absolute pronouncements of Twitter user’s views, but as useful analytical categories to bin groups of Tweets for analysis. Tweets and Twitter user profiles represent only a slice of time, and as our research showed us, Twitter user’s expressed views as well as the content they choose to display on Twitter shift over time. Furthermore, users rarely explicitly expressed their views on all or even any of the slant and bias-related categories for which we coded. The codes and associated rulebook were designed to allow coders to evaluate the ambiguities of Twitter in a consistent, evidence-based, and useful way.

##### Political Slant, Zero-Tolerance Policy Slant, and DNA Testing Slant

For all three categories of slant (named above), a neutral code indicated either an explicitly neutral stance or a lack of evidence to support a code. Key words as well as the source of the code (e.g. text of the Tweet, Twitter user profile) were documented to substantiate any ascribed slant. For political bias, the text of the original Tweet was examined for bias as Democrat, Republican, anti-Democrat, anti-Republican, or neutral. We define anti-Democrat/Republican as any Tweet or Tweeter that “expresses disapproval for the Democratic/Republican party, its head, or multiple members thereof without explicitly expressing approval of another party;” this allowed us to assign slant to a larger number of Tweets and Tweeters without imposing the title of Democrat or Republican where evidence was lacking (see *Codebook*). In addition to the potential slant of the Tweets, the potential bias of the Twitter user was evaluated using these same categories with the addition of the categories of conservative, liberal/progressive, or independent, which were terms used by Twitter users to explicitly express political affiliation. U.S. political terms were used as the events under consideration took place in the geographical and political context of the United States. Determination of a Twitter user’s bias relied upon the profile of the Twitter user and/or prior Tweets by that user. Additional Tweets drawn on to support codes were listed by date under the source code. During reconciliation, coders reviewed each other’s evidence and ran additional searches to finalize decisions on slant and bias data.

##### Combined Coding of Political Slant

After coding both the Tweets and the Twitter users for political slant, we combined the categories for an overall political slant to simplify our analysis. For political slant of the Twitter users, we analyzed the categories separately (i.e., Democrat, Republican, anti-Democrat, anti-Republican, or neutral) and as combined Democrat with anti-Republican and Republican with anti-Democrat. We categorize each Tweet as “neutral” if both the Tweet and the Twitter user codes were neutral. When the Tweet and the Twitter user were coded similarly, we coded them as either “conservative” or “liberal,” as appropriate. For Twitter users that were coded as Independent, we relied upon the code of the Tweet to determine the combined code (see **Supplemental File** “Codebook for Twitter”, **Data Sheet 2**).

### Human Subjects Consideration

No human subjects review was necessary for this research. Great care and thought was given into whether a human subjects exemption was necessary for the online data research, as social media research is still in its early phases (Williams et al., 2017). Given that the retrospective study of published comments (whether in news media or social media) does not involve any intervention, interaction, or observation of public behavior as it was occurring, this research falls outside of §:_104(d)(2) requirements for human subjects research. The revised 2018 Common Rule deems scholarly and journalistic activities (including the collection and use of information that focuses directly on specific individuals) not to be research (U.S. Department of Health and Human Services, 2018).

### Study Limitations

Many challenges accompany research analyzing media—and especially bias in the news media. We focused on traditional newspaper sources for the systematic searches and, when they revealed little substantive content for further analysis, we included a set of articles from non-traditional newspaper locations in an *ad hoc* manner. The resulting news articles seemed to represent a sampling of the types of news media covering the topics of interest. In addition, while we excluded our authored opinion editorial on the topic, we did not exclude news articles for which the authors were consulted or quoted; this could introduce another layer of bias in examining the content, which was why we opted to have a second reader and coder (VO) that was not quoted in any news articles.

Analyzing social media presented a whole other set of challenges. Selecting Twitter as opposed to other social media platforms seemed reasonable and justifiable given the influence of the platform in current politics. The challenge of developing an approach to Twitter data analysis was compounded by the lack of consistent prior approaches for social science research. Our research encompassed evaluation of a niche topic in a specific timeframe, necessitating a novel approach to capture a finite set of Tweets using a set of search strings and criteria, and using these data as a subset example of the Twitter conversations on the topic. Twitter, Inc. faced accusations of exhibiting left-leaning bias due to the disappearance of select conservative Twitter users’ accounts from search suggestions in the summer of 2018 (Selyukh, 2016; Stack, 2018). Since none of the accusing users were in our dataset, and our dataset was relatively small, it is unlikely this affected our data analysis.

Twitter data analysis has specific challenges that we had to address, such as the use of vernacular and casual terms, use of rhetoric and sarcasm, use of political code word or phrases or hashtags, and use of non-textual symbols, images, and emojis. Development of a rulebook and codebook was immensely helpful in evaluating these. We also developed a novel approach of an “interpretation” of each Tweet in order to read each for content without the distraction of the rhetoric and sarcasm that could affect our coding (e.g., “Obama apparently handed out Mexican kids to pedophile traffickers” was interpreted as “Obama administration contributed to trafficking of children”). The interpretation was a successful approach to examine the purpose of each Tweet—for example, to inform *versus* to influence other Twitter users.

## Results

### News Analysis

#### Compilation of Sources Covering DNA in Family Reunification

Prior to initiating our research, we were aware of 39 news articles discussing some aspect of DNA and migrant family reunification. We first conducted a search of the five major dailies (ProQuest search); however, after locating scant coverage, we expanded the search to include other U.S. newspapers (Westlaw Next search) and other news sources (Nexis Uni search) to capture as many relevant news articles as feasible. The three searches for articles using the terms “DNA” and “family reunification” revealed a total of 526 news items, 263 of which were unique (not duplicate) and, of these, 203 of which were relevant to the use of DNA for migrant family reunification (see **Table S1**). Only five of the 39 articles known to the study team were captured in the three searches, so the additional 34 articles were analyzed along with those captured with the systematic searches, resulting in a total of 237 items for consideration. These articles were further refined for eligibility criteria, eliminating 29 items that were TV news transcripts, 21 press releases, 3 articles that were not accessible online, and the opinion editorial authored by the study team (see **Table S2**). This refinement left us with 183 articles from 90 media outlets to read and analyze for depth of coverage.

The potential political slant or bias of the media outlet was assessed in addition to the content analysis of the 183 articles. Three bias assessors were used, and no bias assessor had evaluated the sources of all 183 articles. As shown in **Table 1**, allsides.com had assessed bias on 102 articles at the time of our analysis, revealing six as right-biased, 59 as left-biased, and 37 as center; mediabiasfactcheck.com had assessed bias on 150 articles, revealing 30 as right-biased, 90 left-biased, and 30 as center (termed “least bias” by the mediabiasfactcheck.com); and adfontesmedia.com had assessed bias on 85 articles, revealing 14 as right-biased, 35 as left-biased, and 36 as center (termed “neutral” by adfontesmedia.com) and also revealing 65 as fact reporting, 18 as fair analysis, and two as unfair analysis. As a whole, the majority of media outlets were rated by at least one of the bias assessors as having a left-leaning bias (52.5%, 96/183) (see **Table S3**).

**Table 1:** Media sources and bias.

Source	Number articles mentioning use of DNA (*N* = 183)	Number articles covering DNA with context (*N* = 70)	Number articles addressing science, legality, or ethics (*N* = 27)	Allsides.com bias rating	Mediabiasfactcheck.com bias rating	Adfontesmedia.com
The Washington Post	16	5	1	Left	Left-center	Skews left + fact reporting
The New York Times	10	2	–	Left	Left-center	Neutral + fact reporting
NPR News	8	–	–	Center	Left-center	Neutral + fact reporting
Texas Tribune	8	–	–	–	Least biased	–
The Wall Street Journal	7	1	–	Center	Right-center	Skews right + fact reporting
Associated Press	6	3	2	Center	Least biased	–
Los Angeles Times	6	–	–	Left	Left-center	Skews left + fact reporting
Chicago Tribune	5	1	–	Center	Right-center	–
Politico	5	2	–	Left	Least biased	Neutral + fact reporting
Arkansas Democrat Gazette	4	1	–	–	Right-center	–
Huffington Post	4	–	–	–	Left	Hyper-partisan left + fair analysis
USA Today	4	3	2	Center	Left-center	Neutral + fact reporting
Washington Times	4	2	–	Right	Right-center	Hyper-partisan right + fair analysis
Baltimore Sun	3	2	1	–	Left-center	–
Boston Globe	3	1	1	Left	Left-center	–
Congressional Quarterly News	3	2	–	–	–	–
State Capitol News Feed	3	1	–	–	–	–
Voice of America	3	1	1	–	Least biased	–
Albuquerque Journal	2	1	–	–	Least biased	–
Arizona Republic	2	–	–	–	Right-center	–
Atlantic Online	2	1	1	Left	Left-center	Hyper-partisan left + fair analysis
CNN	2	2	–	Left	Left	Skews left + fair analysis
GenomeWeb	2	2	1	–	–	–
Houston Chronicle	2	1	–	–	Left-center	–
NBC News	2	1	1	Left	Left-center	Neutral + fact reporting
New York Post	2	1	–	Very right	Right-center	Skews right + unfair analysis
Slate	2	1	1	Very left	Left	Hyper-partisan left + fair analysis
Left-leaning sources with one article ^a^	27	21	9	Center Left Very left	Left Left-center	Skews right + fair analysis Skews left + fact reporting Hyper-partisan left + fair analysis Neutral + fact reporting
Right-leaning sources with one article^b^	6	2	2	Center	Right-center	Neutral + fact reporting
Least biased sources with one article^c^	5	4	3	–	Least biased	Neutral + fact reporting
Other sources with one article^d^	25	6	1	–	–	–

a ABC News, Albany Times Union, Arizona Daily Star, BBC News, BuzzFeed, CBS News, CNBC, Electronic Frontier Foundation, Fast Company, Independent, Intercept, Newsday, NY Magazine, Politifact, San Antonio Express-News, San Francisco Chronicle, San Jose Mercury News, Seattle Times, St Paul Pioneer Press, Star Tribune, The Daily Beast, The Hill, The New Republic, The Philadelphia Tribune, The Verge, Time, Wired.

b Daily Herald, Forbes, Fortune, Fort Worth Star Telegram, The Dallas Morning News, The Desert Sun.

c National Geographic, Reuters, Roll Call, San Diego Union Tribune, Scientific American.

d AZ Central, Bucks County Courier Times, Courier Post, Daily Post, Denton Record Chronicle, Federal News Feed, Indy Star, Island Packet, mySA, New Hampshire Union Leader, NewsGram, NorthJersey.com, Salina Journal, Targeted News Service, The Acorn (Drew Univ), The Daily Citizen, The Gainesville Sun, The Joplin Globe, The Keene Sentinel, The Record Herald, The Slot, The Stamford Advocate, US Official News, WGRZ, WorldNet Daily.

**Figure 2:**
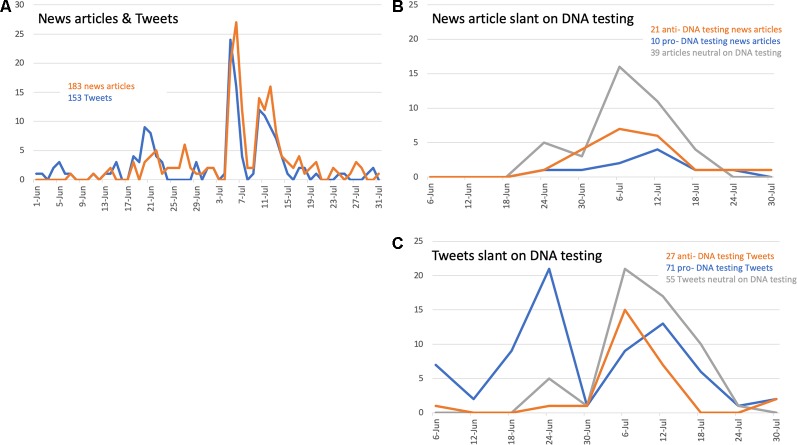
Whole numbers of news articles and Tweets published were binned for 6-day periods to display the trends in publications over the time period of June 1 to July 31, 2018. **(A)** Timeline of news articles and Twitter coverage, showing the overlap in publications in the news with Twitter activity. **(B)** Timeline of slant on DNA testing in news coverage, showing a single peak in the increased number of news articles around July 6, 2018, with the majority being neutral on DNA testing. **(C)** Timeline of slant on DNA testing in Twitter coverage, showing two peaks—one around June 24, 2018 with primarily pro-DNA testing Tweets, and the second around July 6, 2018 with a combination of Tweets that are pro-, anti-, and neutral on DNA testing.

The 183 news items were read by two members of the study team for depth of coverage of the use of DNA. News articles were published sporadically in early June and then nearly every day through July 20 (see **Figure 2A**). There were two dramatic peaks of coverage from July 5 to 7 and again July 10–12 and a minor peak between June 20 and 22. The majority of the 183 articles only mentioned that DNA was being used in the context of family reunification. This refinement for depth of coverage left us with 70 news articles to read and analyze for content and slant (see **Table S4**). These 70 articles covered the period from June 21 to July 28 ranging from 294 to 1,995 words (|equ_0001.eps| = 819, µ = 847) (see **Table S5**).

#### News Slant

In addition to examining the news sources for slant on DNA testing, we examined the articles for their slant on immigration and also on the zero-tolerance policy. Our leading hypothesis for slant coding was that an article could be *for* strong immigration policies but simultaneously *against* the family separation policy that was the by-product of the zero-tolerance policy. Within our dataset, however, we did not find any such instances, so we report here only on the slant regarding the zero-tolerance policy.

In evaluating the news sources for slant on the zero-tolerance policy, we found that the majority of articles were either against the policy (45.7%, 32/70) or neutral (47.1%, 33/70), with only five articles (7.1%, 5/70) containing language demonstrating support for the policy (see **Table S6**). In examining the slant for or against the use of DNA testing in migrant family reunification, we found 10 articles (14.3%, 10/70) to be for the use, 21 (30.0%, 21/70) against, and the majority neutral (55.7%, 39/70) (see **Table S7**). Further examination of the articles with slant on both DNA testing and the zero-tolerance policy shows that half of the 10 articles that were for DNA testing were for the zero-tolerance policy and half against (see **Table 2**). On the other hand, all of the anti-DNA testing articles were either anti-zero-tolerance policy (61.9%, 13/21) or neutral (38.1%, 8/21). All 27 news articles that contained in-depth discussion of science or legal authority were either anti-zero-tolerance policy (44.4%, 12/27) or neutral and either anti-DNA testing (48.1%, 13/27) or neutral. News articles that presented a neutral perspective on both the zero-tolerance policy and on the use of DNA represented 37.1% (26/70) of the total articles. Eight of these articles were included in our list of 27 news articles with more in-depth coverage of science and discussion of legal authority.

**Table 2:** Comparison of zero-tolerance policy slant to DNA testing slant in Tweets and news articles.

DNA testing slant	Number articles (*N* = 70)	Pro-zero-tolerance policy (*N* = 5)	Anti-zero-tolerance policy (*N* = 32)	Neutral on zero-tolerance policy (*N* = 33)
Pro-DNA testing	10	5	5	–
Anti-DNA testing	21	–	14	7
Neutral on DNA testing	39	–	13	26
	**Number Tweets (** ***N*** ** = 153)**	**Pro-zero-tolerance policy (** ***N*** ** = 48)**	**Anti-zero-tolerance policy (** ***N*** ** = 75)**	**Neutral on zero-tolerance policy (** ***N*** ** = 30)**
Pro-DNA testing	71	40 (RT^a^ 10,992)	21 (RT 4,501)	10
Anti-DNA testing	27	–	19 (RT 3,364)	8
Neutral on DNA testing	55	8	35	12

a RT indicates the number of re-Tweets.

#### News Content

While the 70 selected articles covered DNA testing beyond simply mentioning that DNA tests were being conducted, the articles ranged broadly in their scope of coverage. We found that of these 70 articles, only 22 (31.4%, 22/70) mentioned expert consultants in academia (21.4%, 15/70), forensics (2.8%, 2/70), government (17.1%, 12/70), industry (24.3%, 17/70), or a nongovernmental organization (21.4%, 15/70) (see **Table S8**). No law or immigration enforcement authorities were referenced.

The articles covered a range of topics and varying depths with regard to the DNA test application. For instance, many (35.7%, 25/70) specifically covered the use of DNA tests to reunify migrant families following the separation that ended on June 20 (see **Table S9**). Some articles also covered the use of rapid DNA tests at the border prior to separation (8.6%, 6/70), the collection of DNA from immigrant detainees for CODIS as required by 73 FR 74932, 2008 (5.7%, 4/70), and the use of DNA tests for ancestry purposes (7.1%, 5/70). The majority (64.3%, 45/70) were not specific as to how the DNA tests would be applied.

We further examined these 70 news articles for depth of coverage of the science of DNA testing and the legal authority for the DNA testing. We noted that 19 of the articles (27.1%, 19/70) discussed the science enough to warrant further analysis into content and accuracy and that nine (12.9%, 9/70) discussed the legal authority of DNA testing; seven of the articles covered both. An additional six articles did not discuss the science or legal authority for DNA testing; the sole focus of these articles was the ethics of DNA testing. This refinement led to 27 news articles with discussion of DNA testing that warranted further analysis for content. These 27 news articles represent 14.8% of the 183 articles originally identified and eligible for analysis, meaning 85.2% of the news articles covering the use of DNA tests for migrant family reunification did *not* contain any depth of discussion of the science or legal authority for doing so.

#### In-Depth Analysis of News Content

The 27 news articles were further analyzed for coverage regarding the ethics, science, process, and legality of DNA testing in this context (see **Table 3**). In general, of the 27 news articles undergoing in-depth analysis, 13 (48.1%, 13/27) had a slant against DNA testing, and the rest were neutral. Of the 38 topics we assessed, the number covered by news articles ranged from only four topics within the 1,325-word article on July 8 in *The Washington Post* (Sacchetti, 2018c) to 20 topics each in a 1,995-word article on July 5 in *GenomeWeb* (Ray, 2018) and a 1,362-word article on July 10 in *USA Today* (Weise et al., 2018). The number of topics is, of course, less interesting than the scope, depth, and accuracy of coverage, as well as what was not covered by the articles. Our analysis, though not exhaustive, enabled an important glimpse into the substantive quality of the articles when it came to discussion of DNA testing in this realm.

**Table 3:** Topics mentioned in news and on Twitter.

**Topic**	**Subtopic**	**News articles (N = 183)**	**Tweets (N = 153)**
Ethics	Privacy concerns	20 (10.9%)	10 (6.5%)
	Child consent	17 (9.3%)	5 (3.3%)
	Adult consent	15 (8.2%)	3 (2.0%)
	Rights violation	9 (4.9%)	5 (3.3%)
	Language/comprehension barrier	2 (1.1%)	–
	DNA data storage/sharing/destruction	24 (13.1%)	9 (5.9%)
	Unexpected biological families	11 (6.0%)	–
	Non-traditional families	12 (6.6%)	1 (0.7%)
	Vulnerable communities	8 (4.4%)	1 (0.7%)
	Uncovering health information	4 (2.2%)	–
	Uncertainty of who is conducting tests or where tests are performed	9 (4.9%)	4 (2.6%)
	Parents/kids know each other already	2 (1.1%)	1 (0.7%)
	Cultural beliefs against DNA	1 (0.5%)	1 (0.7%)
Science/process	What is DNA	3 (1.6%)	–
	Single nucleotide polymorphisms (SNPs)	2 (1.1%)	–
	Short tandem repeats (STRs)	7 (3.8%)	–
	Rapid DNA testing	7 (3.8%)	–
	What do test results demonstrate	14 (7.7%)	131 (85.6%)
	Commercial DNA test	20 (10.9%)	12 (7.8%)
	Method of DNA collection	19 (10.4%)	–
	Who retrieves specimen	3 (1.6%)	4 (2.6%)
	Time in comparison to other reunification methods	7 (3.8%)	10 (6.5%)
	Time frame of process	7 (3.8%)	3 (2.0%)
	Costs	4 (2.2%)	38 (24.8%)
	Who would pay for testing	10 (5.5%)	45 (29.4%)
	Who would receive the sample report	1 (0.5%)	1 (0.7%)
Legality	Prior use in other immigration programs	9 (4.9%)	3 (2.0%)
	Authority and/or legality of DNA testing	13 (7.1%)	6 (3.9%)
	Court-mandated reunification	10 (5.5%)	9 (5.9%)
	HHS or government DNA testing	11 (6.0%)	68 (44.4%)
	Storage of DNA data in federal immigration database	10 (5.5%)	5 (3.3%)
	DNA for future arrests	10 (5.5%)	2 (1.3%)
	DNA for public safety	–	2 (1.3%)
	DNA for trafficking detection	12 (6.6%)	37 (24.2%)
	DNA to identify undocumented relatives	2 (1.1%)	–
	Oversight	4 (2.2%)	2 (1.3%)
	External/legal advisory for migrants	10 (5.5%)	–
Other	Golden State Killer case	4 (2.2%)	1 (0.7%)

##### Coverage of Science and Processes

With 183 articles mentioning the use of DNA tests, we expected some portion to describe what DNA was or how DNA testing might be applied, and some descriptions of how DNA testing for immigration might differ from other types of DNA tests with which the article’s readers might be familiar (such as DNA ancestry tests). However, only three articles (1.6%, 3/183) had a statement describing DNA, although an additional six mentioned the use of STRs for identity testing. We present examples of quotations from these sources in **Table S10** to demonstrate how genetic concepts was described to the public. Of note, seven articles mentioned the potential use of rapid DNA technologies, albeit with varying depth of coverage.

The articles lacked consistency in describing the DNA testing processes that were being proposed. This lack of clarity was manifested in the articles as inconsistencies in the type of DNA collection that was supposedly performed and also in the anticipated results of the DNA tests. While a majority (55.6%, 15/27) of the articles that mentioned some form of DNA collection by the government indicated that cheek swabs would be performed, some articles instead mentioned the use of saliva (eight articles) or blood samples (two articles). All 13 articles prior to the HHS announcement on July 5 mentioned by name at least one commercial DNA testing service. Of these, six referenced the use of saliva samples, five referenced the use of cheek swabs, and one referenced collection of blood. Given the known processes of the named ancestry companies, the reference to cheek swabs and/or blood was likely an error on the part of the reporters. None of the 13 articles addressed how samples would be retrieved and whether chain-of-custody would be maintained.

The articles discussing DNA testing in June tended to center around the potential use of commercial genetic services and technologies for the reunification of families, whereas the articles in July primarily were focused on the announcement on July 5 that the HHS Office of Refugee Resettlement (ORR) would be using genetic tests as a reunification tool. Seven (3.8%, 7/183) of the articles from July 5 to 31 addressed the uncertainty of what type of DNA testing would be conducted and under what organization or authority the testing would occur. Of the 11 articles that mentioned that the government was conducting DNA tests, only three mentioned who would retrieve the sample and only one article mentioned who would receive the DNA test report.

##### Coverage of Legality

The lack of clarity regarding how DNA testing might be conducted as instructed by the U.S. government was apparent in our analysis of coverage of the legal authority of DNA testing. Many of the articles that expressed concern for the uncertainty of how DNA testing would be conducted also speculated on the legal authority for DNA testing (see **Table S11**). That said, few articles overall (7.1%, 13/183) questioned or considered the legal authority for DNA testing, and these articles were in disagreement as to whether the U.S. government has authority to do so or not. Most of the articles did not mention the need for oversight of genetic testing in this context. Those that did (2.2%, 4/183) did not directly address the need for oversight of DNA testing for immigration or acknowledge the existing oversight mechanisms for relationship testing (see **Table S12**).

Given that the summer 2018 application was not the first time that genetic tests were being used for immigration purposes, we were interested in what the articles covered regarding the prior immigration applications of DNA tests. However, only nine (4.9%, 9/183) news articles mentioned some prior form of government-sponsored DNA testing. Five of these articles had a negative slant on DNA testing, and the rest were neutral. Four of the articles mentioned the voluntary use of DNA tests for verification of family members petitioning to join relatives in the United States; two mentioned the collection of DNA from immigrant detainees as a requirement for CODIS; two mentioned the requirement for DNA tests as part of the affidavit of relationship for Priority-3 refugees joining family members in the United States; two mentioned the use of DNA to screen for claimed relationships of unaccompanied minors coming to the United States to join family members; and one mentioned the DNA-ProKids program that uses DNA tests to detect child trafficking (see **Table S13**).

##### Coverage of Ethics

Several ethics topics of interest were not well represented in the news articles, both in terms of the potentially positive and the potentially negative uses of DNA. For instance, none of the articles addressed the use of DNA for public safety, in as far as the collection of DNA from migrants might be used to protect citizens and legal residents and for future crime solving. Ten (5.5%, 10/183) of the articles did mention the use of DNA for future arrests but only as a negative aspect or as a potential intrusion on the rights of the migrants. Along that vein of potential intrusions, only two (1.1%, 2/183) of the articles mentioned the potential use of DNA to identify other relatives who might be undocumented. Most of the 27 articles addressed at least some of the privacy issues of DNA (20 articles) and/or the concerns for security of the DNA specimen and resulting genetic data (24 articles); however, the 25 articles addressing one or both of these topics only reflects a 13.7% portion of the 183 articles covering DNA testing.

Overall, the articles addressing ethical issues tended to focus on ethical issues commonly associated with health-related genetic testing (e.g., privacy, consent, and storage of information), rather than non-health-related testing (e.g., unexpected kinship, language barriers, differing cultural definitions of family). More articles mentioned consent (10.4%, 19/183), privacy (10.9%, 20/183), or DNA data storage (13.1%, 24/183) than other non-health-related topics. Quite surprisingly, only two articles (1.1%, 2/183) mentioned the risk that migrants could have a language barrier or an inability to comprehend the DNA testing process. Only one article (Richards, 2018) mentioned the potential differences in cultural perspectives on the use of genetic information. Four articles (2.2%, 4/183) mentioned the risk of uncovering or revealing health-related information. How the articles addressed the risks in kinship testing was interesting as well. We compared analysis of coverage of the risk that DNA testing might reveal an unknown biological relationship—or lack thereof (mentioned in 11 articles, 6.0%, 11/183)—to coverage of the interpretation challenge of applying a DNA test to families that are non-biological (mentioned in 12 articles, 6.6%, 12/183). We expected that a news article covering one topic would cover the other, but in fact, only five articles mentioned both.

We found a general lack of discussion of the rights of migrants who could potentially be DNA tested for reunification purposes. Further, of the few articles with some mention of the human rights of the person undergoing the DNA test, six of them (66.7%, 6/9) had a negative slant on DNA, broadly discussing how DNA testing of migrants could be a violation of their human rights. None of the articles addressed whether migrants might have a right to access DNA data in support of their refugee petitions. On a similar note, half of the eight articles that mentioned that migrant communities were vulnerable populations at high risk of being taken advantage of in the DNA testing process had a negative slant on DNA.

Of the 27 news articles, only three lacked any mention of the actor involved in DNA testing (e.g., HHS, commercial genome service provider); offers of commercial DNA tests garnered the most attention, appearing in 20 articles. Articles that mentioned the HHS announcement of DNA testing (following the July 5, 2018 announcement) and those mentioning commercial DNA testing seemed to address ethical topics, covering about 5.3 ethics issues per article (see **Table S14**). Both categories of articles also brought up legality issues—although with less frequency than ethics issues—covering about 3.3 and 2 topics per article, respectively. Within the 13 articles that mentioned commercial DNA testing, some mentioned the method of collection (61.5%, 8/13) and some mentioned who would pay for testing (69.2%, 9/13). In contrast, the four articles that mentioned only the HHS announcement of DNA testing contained no mention of the issue of who would pay for testing. All seven articles that mentioned both the HHS announcement of DNA testing and commercial DNA testing mentioned method of collection, but surprisingly, only one article (14.3%, 1/7) mentioned the issue of who would pay for testing.

Another theme among the 27 news articles was discussion of potential secondary uses of genetic data collected from migrants, mentioned in 18 news articles. These topics include using DNA (either DNA testing or DNA collection) for detecting human trafficking, apprehending wanted criminals, solving future crimes, and identifying undocumented relatives. Using DNA for trafficking detection was mentioned in 12 articles, and using DNA for future arrests was mentioned in 10 articles. No trends were seen in slant for or against DNA testing in these two sets of articles. However, only two articles mentioned using DNA testing for identifying undocumented relatives, both of which were coded as negatively slanted against DNA. No articles mentioned the use of DNA to apprehend wanted criminals among the migrating population.

Finally, we were interested in examining the relevancy of other topics mentioned in the course of the news articles focusing on DNA testing, as we anticipated the potential for irrelevant topics to be included as sensationalism (whether intentionally or unintentionally). One topic we tested as a measure of this was the recent use of familial searching of genealogical databases for crime investigations, as was used in the Golden State Killer case in April 2018. This case was mentioned in four of the news articles, all of which had a neutral stance on DNA testing for family reunification.

### Twitter Analysis

#### Searches for Coverage of DNA in Family Reunification

The search for “DNA” with trending hashtags yielded 59 Tweets and the search of “DNA” with key words yielded 164 Tweets (see **Table S15**); four of the Tweets were in both searches. One Tweet in each search were duplicates, so only the older Tweet was kept for data analysis. While the PDF used to capture Tweets during our one-time search contained hyperlinks to the source Tweets (allowing us to view images and other data such as exact time of posting and geolocation), we found over time that some Tweets became later inaccessible; we encountered problems such as the suspension of accounts and changes in display or usernames. We found that conducting searches for specific Tweets using the user interface did not consistently return relevant results. The Tweets were refined for relevancy to the topic of migrant family reunification resulting in 153 Tweets for further analysis. The trends in timing of Tweets parallels those trends observed in the news coverage, again with two peaks in conversation from July 5 to 6 and again July 10–11, and a minor peak between June 20 and 22 (see **Figure 2A**).

Of the all of the Tweets in our dataset, 133 (86.9%, 133/153) were original Tweets and 20 (13.1%, 20/153) were re-Tweets. In 24 Tweets (15.7%, 24/153) other Twitter handles were directly mentioned (e.g., @POTUS, @DHS), and 84 Tweets (54.9%, 84/153) contained hyperlinks to news articles (see **Table S16**). In 26 Tweets (31.0%, 26/84), the news articles were 12 of those included in our news source analysis (two Nexis Uni + Westlaw Next; one Nexis Uni + ProQuest; nine *ad hoc*). The other news articles hyperlinked to Tweets were articles not specifically discussing DNA testing but rather the border crisis in general. Imagery was used in many of the Tweets, not only through the hyperlinked articles but also with in the form of memes, statistics, or videos in 15 Tweets (9.8%, 15/153) and in the form of emojis in 10 Tweets (6.5%, 10/153).

#### Hashtags and Direct Mentions in Tweets

Seasoned Twitter users use hashtags (#) and direct mentions (@) in their Tweets to target specific topics or other Twitter users, respectively. Among our 153 Tweets, 60 (39.2%, 60/153) used hashtags, and 26 Tweets (17.0%, 26/153) used direct mentions. We grouped the hashtags into themes and tabulated the number of Tweets and re-Tweets of these themes to assess the effects of the hashtags on the spread of conversations (see **Table S17**). One of our two searches to capture data for analysis used hashtags as part of the search string, so it was unsurprising that these 17 hashtags were detected. Nevertheless, these and the other resulting hashtags were useful in qualitatively examining the effects of different hashtag terms on re-Tweets. Direct mentions in our data set were similarly evaluated and grouped by category of the user. These data show the intended audience for at least some of the ongoing Twitter conversations (see **Table S18**).

#### Purpose of Tweets and Common Language

As a novel approach to analyzing the content of the Tweets, we developed an interpretation of each Tweet as outlined in the *Materials and Methods* section. Using these interpretations, we were able to break down the Tweet to code for its intended purpose using the verbs selected for the interpretation, including Tweets that advocate, announce, call upon, comment, express, hint, question, report, share, solicit, or suggest. Some Tweets have multiple purposes (see **Table S19**). While interpreting the Tweets allowed us to clarify content and check our own biases in interpreting the message of a Tweet, the rhetoric used in the original Tweet was key for coding for slant.

#### Tweet Slant

Support for the zero-tolerance policy, not always explicitly named by Twitter users, was associated with support for stricter enforcement of immigration laws. Support for family separation was associated with support for the zero-tolerance policy, but an anti-family separation stance was not assumed to indicate an anti-zero tolerance policy stance. Coding for DNA testing slant was relatively unproblematic as long as coders had a rubric to distinguish between factual reporting and expressions of support. Political biases of Tweet and Twitter users depended on expressions of support for or affiliation with a party, its head, or multiple party members; this could be in the form of text, including hashtags and direct mentions, as well as images (especially emojis associated with particular parties, such as the “blue wave” emoji). Where Twitter users explicitly identified themselves as belonging to a particular political group (e.g., conservative, independent), we adopted their terminology so as not to impose an over-simplified coding scheme on the complex political landscape visible on Twitter.

The Twitter traffic on the zero-tolerance policy and DNA testing contrasted with the news articles to some degree. Like the news articles, we presumed a Twitter user could, in theory, be supportive of strong immigration policies but opposed to the family separation policy that results from the zero-tolerance policy. Given the brevity of Tweets, though, it was impossible to code for stance on immigration in general as we had done in the analysis of news articles. However, we could code for political leanings of the Tweet itself and the Twitter user, in addition to a Tweet’s stance on the zero-tolerance policy. We found just over half of Tweets were neutral (51.0%, 78/153), 45 (29.4%, 45/153) had a Democrat or anti-Republican slant, and 30 (19.6%, 30/153) had a Republican or anti-Democrat slant (see **Table 4**).

**Table 4:** Political slant on Twitter and impact as measured by re-Tweets.

	**Number Tweets**	**Total re-Tweets**
**Political slant of Tweets**		
Republican	21 (13.7%)	8,318 (26.0%)
Anti-Democrat	9 (5.9%)	1,382 (4.3%)
Democrat	3 (2.0%)	85 (0.3%)
Anti-Republican	42 (27.5%)	7,813 (24.4%)
Neutral	78 (51.0%)	14,378 (45.0%)
**Political slant of Twitter users**		
Republican	40 (26.1%)	11,045 (34.5%)
Anti-Democrat	–	–
Conservative	13 (8.5%)	1,762 (5.5%)
Democrat	39 (25.5%)	3,841 (21.2%)
Anti-Republican	28 (18.3%)	4,468 (14.0%)
Liberal/progressive	8 (5.2%)	570 (1.8%)
Independent	6 (3.9%)	3,520 (11.0%)
Neutral	19 (12.4%)	3,841 (12.0%)
**Combined political slant of Tweet and Twitter users**		
Conservative	52 (34.0%)	12,807 (40.1%)
Liberal	80 (52.3%)	16,508 (51.6%)
Ambiguous	1 (0.7%)	–
Neutral	20 (13.1%)	2,661 (8.3%)

**Figure 3:**
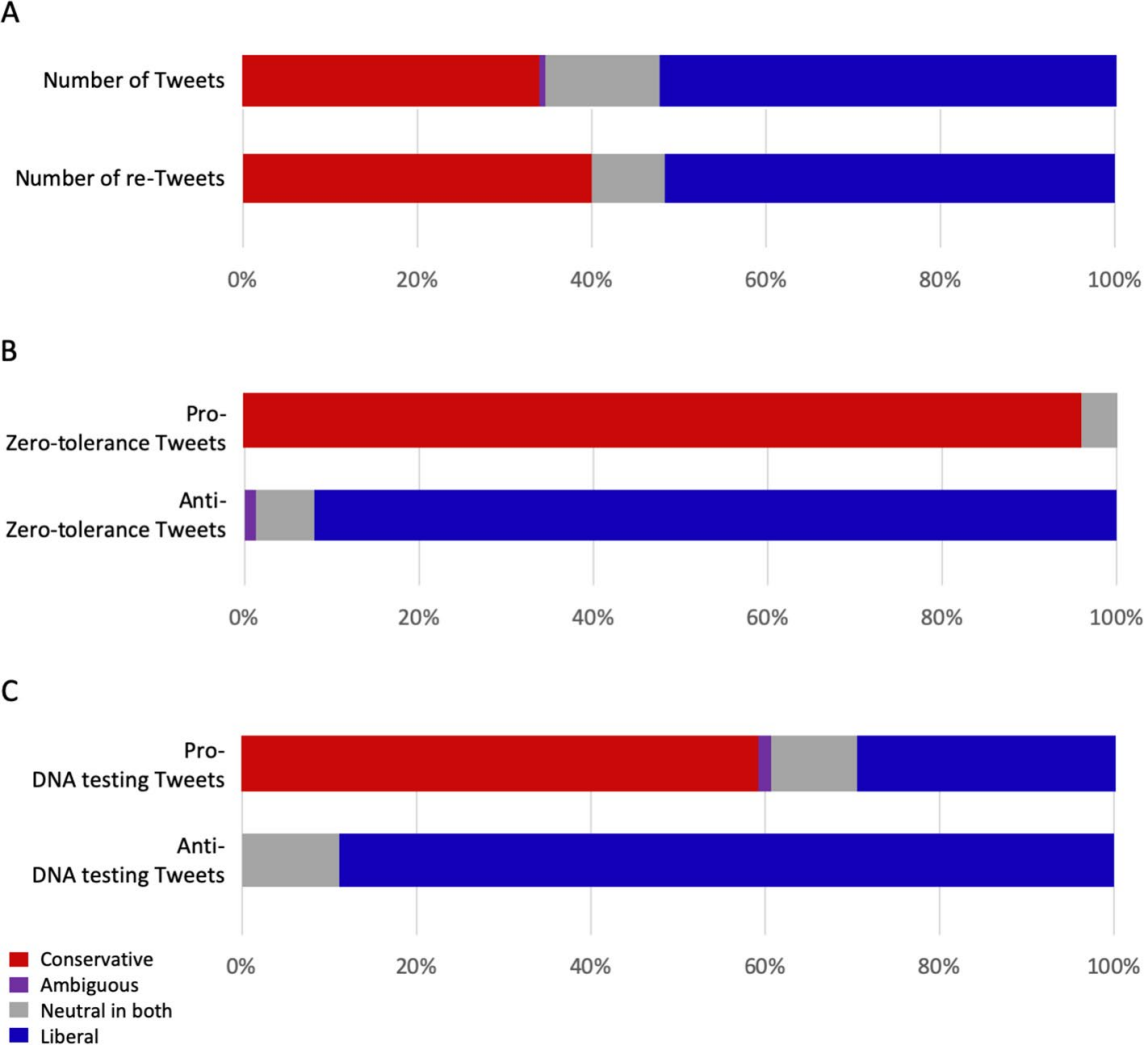
Political slant of Tweets. **(A)** Political slant of each Tweet was evaluated independently of political slant of the Twitter user for each Tweet (see **Figure S1**), then combined to simplify further analysis. **(B)** Analysis of zero-tolerance policy slant of Tweets, showing political partisanship. **(C)** Analysis of DNA testing slant of Tweets showing skewing of slant on DNA testing among political groups.

The combined analysis of political slant of Tweets and Twitter users was fairly consistent in the codes, with only one coded as “ambiguous” (wherein the Tweet was coded as anti-Republican but the Twitter user coded as Republican). In addition to examining the whole counts of each political category, we calculated the number of re-Tweets for each Tweet as a proxy for impact of the Tweet. Ultimately, a combined analysis of the political slant of the Tweet and Twitter user together showed no particular weight of any political grouping (see **Table 4**, **Figure 3A**). We found that the levels of engagement across Tweets as measured by re-Tweets were similar regardless of partisanship (see **Supplemental File** “*Supplemental Tables*”).

The positions on zero-tolerance policy were generally consistent with the expectation that conservatives would support the policy and liberals would oppose the policy. We found that 46 (88.5%, 46/52) conservative Tweets were pro-zero-tolerance policy, with the other six being neutral (see **Table S20**, **Figure 3B**). The liberal Tweets also were consistent, with 69 (86.3%, 69/80) being anti-zero-tolerance policy and the remaining 11 neutral. The majority of neutral Tweets were also neutral on the zero-tolerance policy (65.0%, 13/20).

The slant on DNA testing also showed some interesting bias. Of the conservatives, 42 (80.8%, 42/52) were pro-DNA testing, with the remaining 10 being neutral (see **Table S21**, **Figure 3C**). The liberal Tweets, however, were diverse with regard to slant toward DNA testing, with 21 (26.3%, 21/80) pro-DNA testing, 24 (30.0%, 24/80) anti-DNA testing, and 35 (43.8%, 35/80) neutral. Half of the politically neutral Tweets (in the combined analysis) were neutral on DNA testing. Of particular note, all of the pro-zero-tolerance policy Tweets were also either pro-DNA testing or neutral, whereas the anti-zero-tolerance policy Tweets were split among the three categories (see **Table 2**).

#### Tweet Content and Concepts Covered

In contrast to the 27 news articles evaluated for content from journalists’ perspectives, the set of 153 Tweets reflect the real-time public dialogue as the news was emerging. The content of the Tweets, therefore, overlapped with that of the 27 news articles, but the themes were slightly different (see **Table 3**). Many of the Tweets directly referenced a public figure or organization within the text or through a direct mention (see **Table S22**).

The Tweets ranged in content, but some clear themes emerged, including direct reference to or understanding of what DNA tests would demonstrate, which was apparent in 131 Tweets (85.6%, 131/153). A total of 90 Tweets (58.8%, 90/153) referenced the use of DNA tests to demonstrate parent-child relationships, and an additional 39 Tweets (25.5%, 39/153) referenced use of DNA for general relationship testing. The use of DNA tests for ancestry came up in seven Tweets (4.6%, 7/153), five of which had already mentioned the use for testing kinship or parentage. No Tweets mentioned the potential detection of health-related information. Another emergent theme was concerns about costs of DNA tests and who would bear the financial burdens. Forty-eight Tweets (31.4%, 48/153) mentioned one or both of these related topics. This particular topic (the burden of cost of DNA tests) was a point of discussion almost entirely among politically slanted liberal or neutral Twitter users, with only one Tweet on this point from a politically conservative Twitter user. The majority were against the zero-tolerance policy, while perspectives on DNA testing were more evenly spread. Twitter conversations regarding cost ranged from issues of families reportedly having to pay for their own DNA tests, offers of free DNA testing services, and potential costs to the government and taxpayers. Cost in terms of specific dollar amounts was covered in 12 (7.8%, 12/153) Tweets and offers of free services (including pro bono work or reimbursement for migrant families) were discussed in 6 (3.9%, 6/153) Tweets. Two (1.3%, 2/153) Tweets asserted that the government should pay for DNA tests and one (0.7%, 1/153) asserted that an NGO should pay, while 12 (7.8%, 12/153) asserted that a company (e.g., 23andMe) could pay. Thirty Tweets (19.6%, 30/153) discussed that migrant families had to pay for DNA tests and 26 (17.0%, 26/153) Tweets framed the cost of DNA testing as a burden on migrant families (see **Table S23**).

Unlike the news articles, in which human trafficking was rarely mentioned in 6.6% (12/183) of the articles, 44% (68/153) of the Tweets acknowledged the use of DNA testing to detect human trafficking (see **Table S24**). All but one of these Tweets were from conservative or neutral Twitter users that were supportive of the zero-tolerance policy and the use of DNA testing for reunification. While the overwhelming majority of Tweets that addressed applications of DNA tests beyond family reunification discussed human trafficking, the use of DNA samples or DNA test results, or access to DNA data through a database (e.g., CODIS) for future arrests was addressed in only two (1.3%, 2/153) Tweets, both with an anti-DNA testing slant. Two Tweets also covered the use of DNA samples or DNA test results for detecting unwanted criminals among migrants (one was neutral on DNA testing, the other pro-DNA testing). Discussion of the use of DNA to detect undocumented relatives of migrants was conspicuously absent.

#### Timeline of Slant in News and Twitter Coverage

Because the majority of news articles did not contain enough substance on DNA testing to determine whether there was slant, the timeline of the slant on the news articles was unremarkable. The pro-, anti-, and neutral DNA testing coverage in the news articles was sporadic across the time period (see **Figure 2B**). However, the trend seen in the Twitter timeline differed from the news article trends (see **Figure 2C**). We found that the Tweets published in June were primarily pro-DNA testing, whereas the Tweets in July (following the HHS announcement on July 5, 2018), were neutral, anti-DNA testing and pro-DNA testing.

## Discussion

News on the zero-tolerance policy did not gain a great amount of public attention until the end of May 2018. By mid-June, the public media sphere was largely focused on the policy as an immigration deterrence tool, the ethics of family separations, and backlash rhetoric aimed at the policymakers. It was during this news spiral that DNA testing emerged as a human rights tool for detecting human trafficking at the border, as a reparative tool for reunifying families, and as a screening tool for ensuring children were returned to their rightful relatives. In real time, and anecdotally, the media conversations at large seemed confused in facts and rife with hyperbole. Given the prominent rhetoric and partisanship, we took this opportunity to examine the microcosm of conversations around DNA testing in this context to assess the accuracy of the information being conveyed and bias of the opinions espoused. Our analysis of a subset of news articles and Tweets cannot provide a generalizable understanding of the public’s perceptions of DNA testing, but this evaluation does serve to understand a sampling of the public’s perspectives regarding DNA testing in this specific immigration and border context (family reunification). We hoped that our examination of this current event might be useful for understanding part of what was conveyed and understood about DNA testing in the media to inform future science communication on similar topics of interest and import to the broader public.

While we captured few inaccuracies in either media type, we did note a few examples of misconceptions and misunderstandings of how DNA testing works, especially in the proposed contexts (immigration as opposed to medical testing). For instance, in news coverage we noted two articles discussing the use of blood for DNA tests as well as occasional conflation of saliva samples *versus* buccal swabs for HHS DNA tests and commercial DNA tests. Alarmingly, most of the articles covering DNA testing in family reunification seemed unaware that DNA testing was a routine (sometimes recommended) voluntary metric for immigration petitions, required in certain refugee cases, and routinely collected by law from federal immigrant detainees for the federal criminal database, CODIS. Because of this, the rhetoric that was used in a majority of news articles made it appear that government-mandated DNA tests was a new phenomenon instead of a method that had been previously applied in other similar situations. This can affect the public’s formation of opinions on DNA testing and can lead to inaccurate understandings of how DNA testing had already been used and its previously acknowledged benefits for immigration use.

Most alarming was the fact that the majority of news articles had to be excluded from our content analysis entirely because they omitted any coverage of the purpose of a DNA test, the science behind a DNA test, or what a DNA test might reveal in this non-medical context. Given the persistent misconceptions among the public regarding how DNA tests were conducted and the risks and benefits of a DNA test, adequate coverage of basic science facts (such as defining DNA testing or distinguishing types of testing) is essential for the public to grasp and appreciate the social and policy issues at hand.

Surprisingly, when it came to the purpose of DNA tests, the Tweets contained more nuance than the news articles. While the character count is limited in a Tweet, the majority of the Tweets mentioned in some form that the DNA tests were for parentage. The Twitter conversations also had far more coverage of the potential costs of DNA testing to the government and to the families being tested. While cost and payment were covered in 2.2 and 5.5% of news articles, respectively, these were points made in 24.8 and 29.4% of Tweets, respectively.

We also noticed that the few news articles examining the ethical issues with DNA testing a migrant population—including migrant minors—selectively covered certain potential ethical issues. None of the 10 news articles coded as pro-DNA testing covered these topics. In the anti-DNA and neutral articles, coverage was lacking and inconsistent for two particular topics: 1) the risk of detecting misattributed parentage; and 2) the false equivalency of biological family with the social construct of family (Lee and Voigt, 2019). We were surprised that only 5 of the news articles discussing the former also mentioned the latter and that 11 articles mentioned one topic and not the other. Furthermore, except for covering issues of costs and burden of DNA tests, few of the Tweets referenced ethical issues.

There was no conversation in our dataset about the use of genetic information potentially being an empowering tool for migrants, especially as a means to reunify with their families. The positive discussions around DNA testing were more skewed toward the tool as a means to screen bad actors (i.e., human traffickers) rather than a tool to expedite the reunification processes. While several articles implied that the use of DNA tests might be a ruse to collect DNA from migrants for a database—such as a database for detecting recidivism in illegal crossings or for identifying future criminals—this was not discussed explicitly in any of the articles. The potential use of DNA tests to screen for fraudulent relationship claims (potentially to detect cases of human trafficking) was sparsely mentioned in 6.6% of the news articles but mentioned in 24.2% of the Tweets.

There are dangers in transferring general notions about informed consent in a medical setting, for example with this specific non-medical application of DNA testing. Wholly absent from the public discourse in these news articles and Tweets was a weighing of risks and benefits. While contextual vulnerabilities of the detained migrants now separated from family members should be considered an essential aspect of the ethical and policy inquiry as to whether DNA testing is appropriate (e.g., adequacy of consent, consideration of privacy measures), so too is consideration of the potential direct benefits to those specific individuals (i.e., mitigation of the psychological trauma that could result from indefinite delays in familial reunifications). We agree that genetic information might reveal private information (Katsanis et al., 2018), and that the use of DNA data should be restricted (Lee and Voigt, 2019), but the informational risks need to be balanced with the risks to the families in this context in order to prioritize the reunification of children with their family members expeditiously.

It is critical for genetic professionals and the public to keep distinct conversations on social implications for different types of DNA testing. For instance, DNA testing is not equivalent to DNA collection, since the former might or might not involve database development and the latter might or might not involve a DNA test report. These different uses of DNA have very different social implications. In summer 2018, conversations on DNA testing for family reunification were conflated with conversations about investigative genetic genealogy. It so happened that the timing of the first cases of the use of genetic genealogy to investigate cold cases was a news highlight parallel to the use of DNA for migrant family reunifications. Yet again, the social implications of the two topics barely overlap, except that they both involve DNA and governmental actors. But 4 of the 27 articles in our substantive dataset mentioned the “Golden State Killer.” We assert that these mentions are demonstrative of a) the conflation of discrete nonmedical DNA test issues among the public; and b) the intent of the news media to sensationalize the use of nonmedical DNA tests. Each application has its own implications deserving of public scrutiny and debate.

The greatest challenge to detangling the public dialogue around the use of DNA tests by ORR was the lack of facts and information regarding how DNA testing was being conducted, whether DNA testing was being done, whether DNA was being collected for future use, and what actors were involved in the DNA testing processes. The ORR was emphatically silent on naming the contract laboratory that would supposedly perform the DNA tests, so experts consulted by the media had no choice but to speculate and rightfully raised more questions than answers. This lack of transparency served to spark controversy where facts otherwise might have pacified readers regarding oversight measures for quality, best practice restrictions on secondary uses, or requirements for informed consent. As a result, our data exemplifies the confusion in the media reports and the multitude of questions from the public appearing on social media. Whereas on Twitter we might expect confusion (because information is essentially crowdsourced), the news media has a professional responsibility to provide the public with reliable information. Scientists, too, have a responsibility to share their expertise with the public and a responsibility to foster public understanding. Genetics experts have a responsibility to “claim expertise only in fields where they have the necessary depth of knowledge, especially when interacting with patients, or contributing to public discussion or policy debate” (ASHG, 2017) and to “serve as a source of reliable information and expert opinion” (NSGC, 2017). In summer 2018, the genetics community did not adequately clarify to the public how the science could by appropriately applied—especially in this case of the endangerment of a person’s well-being or a violation of their human rights.

Nearly three dozen members of Congress, led by Representative Jackie Speier (D-CA-14), to write HHS Secretary Azar seeking answers to eight questions surrounding the DNA testing and use of the resulting genetic information. Separately, an amendment introduced by Representatives Clark and Kaptur was adopted in committee markup for the Labor-HHS-Education Appropriations minibus bill to impose restrictions on use and protect the privacy of the genetic information obtained in this context. This amendment was included in the final version of the appropriations bill (H.R. 6157) that became law (the Department of Defense and Labor, HHS, and Education Appropriations Act, 2019 and Continuing Appropriations Act, 2019; Pub. L. 115–245) on September 28, 2018. However, as of April 2019, HHS Secretary Azar had not yet submitted a response to the Congressional letter.

Political slant is expected in any democratic public dialogue, and our data reflected the opinions, innovations, and freedom of thought and press that we expect in a democracy. What was unexpected was to see the political slant (conservative *vs.* liberal) correlating to the slant in opposition to the use of DNA testing. Moreover, we were struck by how the dialogue shifted among liberals in our data set after the announcement that HHS would be using DNA tests for reunification. In fact, we noted three different conversations in the media and on social media: 1) use DNA to detect child trafficking; 2) use DNA to reunify families; and 3) don’t use DNA tests because it is a privacy risk. Conversation #1 was absent from discussion among liberals in our data, and conversations #2 and #3 were absent among conservatives in our data. Interestingly, conversation #2 occurred in regard to commercial DNA tests and conversation #3 occurred in regard to ORR-directed DNA tests. The contrasts and political leanings among these three different conversations exemplify the divisiveness of the public’s conversations about immigration and their understanding of how genetic tools might apply.

Our study of news and social media highlights the gaps in understanding of the public and of the genetics community in how genetic information is routinely used in immigration already. Professional organizations have an important role in communicating with and translating for the public advancements in science and their limits, possibilities, benefits, and risks. American College of Medical Genetics, American Society of Human Genetics, and the American College of Physicians were among the numerous professional organizations rightfully responsive to the summer 2018 crisis, stating their positions in opposition to government-sanctioned activities and recommendations for improvements in policies (American College of Physicians, 2018, American College of Medical Genetics and Genomics, 2018; Nelson, 2018a; Nelson, 2018b). However, the nuances with regard to the prior use of DNA tests in immigration and the existing oversight mechanisms were absent from these statements. So too was a nuanced perspective on the ethics regarding DNA testing given the crisis scenario at that time. We have learned through our research with human trafficking victims and other vulnerable populations (Katsanis et al., 2017) that perspectives on the risks of the use of genetic information is heavily dependent on the context and urgency of that use. Professional organizations should take a proactive role in developing resources applicable in times of crisis to manage misinformation with regard to what genetic information can and cannot convey, and how genetic information might be used and protected. As part of this preparedness, professional organizations should be the point resource for the public in providing relevant expertise so that disseminated content is responsibly responsive.

## Conclusion

The confusion of the news media and reflected in social media on how DNA tests work and the different contexts for applying DNA testing exemplifies A) the lack of transparency in use of genetic information; B) the ineffectiveness of inclusion of genetics experts in the public conversations; and C) the ill-preparedness of the genetics community in reacting to public confusion and outcry. Responsible use of genetic information as a tool in human rights crisis has the potential to garner broader public support for genetic testing in other spaces, but the lack of transparency in how the DNA testing was applied only undermines the trustworthiness of HHS as an institution and HHS-funded genetic research more broadly.

One clear need is improved science communication among our genetics trainees and opportunities for journalists to gain a foundational training in genetics. Such training could improve the overall communication efforts and misunderstandings when it comes to genetic testing. Professional organizations also could develop repositories of information briefs and contacts for relevant experts, so that they are available for news reporters who need rapid and consistent answers when unexpected uses of genetic information emerge.

In general, this study serves as a reminder that the content of genetic experts’ conversations in public spaces are essential in formulating public opinion and communicating the risks and benefits of DNA testing.

## Data Availability Statement

Raw data containing identifiers (e.g., Twitter user names) are not available for public review. All other data are available upon request to the corresponding author.

## Author Contributions

JW and SK conceived of and led the research study. JW and SK conducted the news source searches. All authors analyzed news source content. DM and SK conducted the Twitter searches and analyzed the Twitter content. All authors provided critical feedback and helped shape the research, analysis and manuscript.

## Funding

SK, VO, and JW are supported in part by Grant No. R01HG009923 from the NHGRI. JW’s contribution was supported in part by Grant No. R00G006446 from the NHGRI.

## Conflict of Interest

The authors declare that the research was conducted in the absence of any commercial or financial relationships that could be construed as a potential conflict of interest.
