# Supplementary material for: Conversations Surrounding the Use of DNA Tests in the Family Reunification of Migrants Separated at the United States-Mexico Border in 2018
Source: Front Genet. 2019 Dec 13;10:1232. doi: 10.3389/fgene.2019.01232 (PMC6927295; doi:10.3389/fgene.2019.01232)
Supplement: Supplementary file 1 [file DataSheet_1.docx]

**CODEBOOK FOR 183 NEWS SOURCES**

**COVERING DNA AND FAMILY REUNIFICATION**

**FROM JUNE 1 – JULY 31, 2018**

**CODING PROCESS**

**Two coders separately:**

1. Read or review the main text of the news source. Note terms or excerpts to define any keywords that might indicate slant.
2. Document codes as described below.

**The two coders reconcile their independent evaluation of the news source through discussion.**

**GENERAL INFORMATION**

| Article # | Assigned search source (could be multiple search sources, only one assigned for record naming) |
| --- | --- |
| Full citation |  |
| Article title | Title |
| Article source | News source |
| Author(s) | Last, First |
| Date published | Month Day, Year |

**INITIAL REVIEW**

| Inclusion / exclusion criteria | Include or  Exclude: lack of DNA discussion or duplicate record |
| --- | --- |
| Article type | Opinion editorial  Journalistic article |
| Word count | Number of words excluding figure legends, titles, and subtitles |
| Science discussion | Yes: discussion of DNA testing  No: no discussion of the science of DNA testing  Focus: no scientific discussion, but article’s focus is on DNA testing |
| Legal reference | Yes: discussion of the legal authority for DNA testing  No: no discussion of the legal authority for DNA testing |

**SLANT**

| Immigration | Pro/con/neutral: article has a slant for or against immigration or immigrants, legal or otherwise (e.g., keywords include “illegal immigrant” and “build a wall”) |
| --- | --- |
| Zero-tolerance policy | Pro/con/neutral: article has a slant for or against the Trump administration’s zero-tolerance policy prosecuting illegal border crossers and asylum seekers as criminals |
| DNA | Pro/con/neutral: article has a slant for or against the use of DNA in the context of migration and family reunification |

**INITIAL CONTENT EVALUATION**

| Experts consulted | A: academic  F: forensic  G: government  P: police  I: industry  C: charity  NA: not applicable |
| --- | --- |
| Topics covered | D: detainee DNA collection  B: at-border family DNA testing  R: post-border DNA testing for family reunification  A: ancestry DNA testing. |
