# Supplementary material for: Conversations Surrounding the Use of DNA Tests in the Family Reunification of Migrants Separated at the United States-Mexico Border in 2018
Source: Front Genet. 2019 Dec 13;10:1232. doi: 10.3389/fgene.2019.01232 (PMC6927295; doi:10.3389/fgene.2019.01232)
Supplement: Supplementary file 2 [file DataSheet_2.docx]

**CODEBOOK AND RULEBOOK FOR 153 TWEETS COVERING DNA AND FAMILY REUNIFICATION FROM JUNE 1 – JULY 31, 2018**

**CODING PROCESS**

**Two coders separately:**

1. Review the main text of the Tweet, including emojis. Note terms to define any keywords that might indicate slant.
2. Review any images and additional text, such as headlines of hyperlinked articles, visible in the Tweet.
3. If the Tweet is a reply, re-Tweet, or part of a thread, review all connected Tweets.
4. Review content hyperlinked in Tweet.
5. Interpret the content of the Tweet with the aid of the data reviewed in steps 1-4.
   1. Use full sentences.
   2. Describe the content using neutral language.
   3. Use verbs to describe the intention of the Twitter user (e.g., comment, share).

**The two coders reconcile their independent interpretations of the Tweet through discussion. The two coders then separately:**

1. Code for purpose using the verbs selected for the final Tweet interpretation.
2. Code for ethics discussion.
3. Code for all Tweet characteristics.
4. Code for slant:
   1. Assign codes for political slant of Tweet and Twitter user, pro-/anti- zero-tolerance, and pro-/anti- DNA testing if possible.
   2. If not possible, go on to review, in the following order:
      1. the profile of the Twitter user, including any hyperlinks in the profile.
      2. at least the first 25 of the Twitter user’s other Tweets as displayed in the timeline and media sections of the user’s profile.
   3. Search Twitter for the Twitter user’s username AND relevant keywords.
      1. Searches must be balanced (e.g., if a search is run for “username AND Democrat,” one must be run for “username AND Republican,” along with other possibilities).
      2. Record the date of any Tweets that support a code under the source column and screenshot them.
   4. Search the internet for the individual or organization behind the Twitter account.
      1. If the organization is a news outlet, use a bias checker to confirm political slant.
      2. Review content linked in Tweet in more depth.

**GENERAL RULES**

**Coders will observe the following rules for working with Twitter data:**

1. Use the text of the Tweet itself to code for slant. The interpretation clarifies content but does not help interpret bias.
2. Hashtags are to be interpreted in context and not automatically assumed to indicate affiliation with or support for a group or cause.
   1. If the meaning of a hashtag or emoji is unclear, define by: (a) conducting a Twitter search to view it in the context of other tweets; and/or (b) conducting an internet search.
3. The intent of content sharing (e.g., re-Tweets or attached documents, images) is to be evaluated in the context of the Tweet/Twitter user where possible.
   1. In instances where content is shared with no comment, support for the content is generally assumed.
4. While the most weight is given to text of the Tweet, images (e.g., emojis, memes, photographs) may also contribute to slant codes.

**TWEET CHARACTERISTICS**

| **CHARACTERISTIC** | **CODE** | **DESCRIPTION** |
| --- | --- | --- |
| Username | @text | The unique ID assigned to the Twitter user, always preceded by the @ symbol* |
| Display Name | Text | The name chosen by the Twitter user to identify their account to readers. Displayed next to the username* |
| Date Posted | MM/DD/YYYY | Date the Tweet was posted |
| Number of Comments | # | Number of comments on the Tweet |
| Number of re-Tweets | # | Number of times the Tweet has been re-published by Twitter users |
| Number of Likes | # | Number of Twitter users who have indicated approval of the Tweet through the “like” button |
| Emojis in Display Name | Yes or No | Small images sometimes included in display name to express identity, subtilties of tone, or group affiliation* |
| Emojis in Text | Yes or No | Small images sometimes included in the text of the Tweet to express identity, subtilties of tone, or group affiliation |
| Re-Tweet | Yes or No | Is the Tweet a re-Tweet (a re-publication of another Twitter-user’s Tweet, with or without original commentary) |
| Reply | Yes or No | Is the Tweet a reply to another Tweet |
| Thread | Yes or No | Is the Tweet part of a thread (a series of Tweets linked together by the Twitter user) |
| Attachments | Text or No | Materials attached to the Tweet [e.g., images, links] |
| Direct Mentions | @text or No | Inclusion of usernames of other Twitter users in the text of the Tweet (“direct mentions”), used to highlight another Twitter user or to call the Tweet to the attention of a particular Twitter-user |
| Hashtags | #text or No | Inclusion of succinct phrases preceded by the # symbol, used to join an ongoing dialogue on Twitter or express affiliation with a group or cause |
| Searchable on Twitter as of December 2018 | Yes or No | Can the Tweet be found through a simple Twitter search as of December 2018 |
| Search | Search A or Search B | Which of two one-time searches pulled the Tweet, Search A (DNA plus 17 trending hashtags) or Search B (DNA plus five terms relevant to migration) |
| N# of attachments | N# or n/a | The letter / number combination we assigned to the newspaper articles collected for the news analysis, if the Tweet contains a link to one of these articles |

*Can be changed by user.

**CONTENT: TWEETS ONLY**

See “Codebook for News and Twitter Content” for content codes applied to both Tweets and newspaper articles.

| **ASPECT OF CONTENT** | **CODE** | **DESCRIPTION** |
| --- | --- | --- |
| Purpose | advocates, announces, calls on, comments, expresses, questions, reports, shares, solicits, suggests, hints | Draws on verbs used in the translation of the Tweet to characterize the intention of the Twitter user |
| Ethics Mentions | Yes or No | Inclusion of mention of the ethical challenges of using DNA testing to reunite families |

**Rules for coding slant**

- Separate coding of political slant of Tweet and/or Twitter user from the coding of the politically-charged zero-tolerance policy.

***Political Slant of Tweet and/or Twitter user***

- Focus primarily on the aspects of a Tweet that are visible to a user scrolling through a timeline (a chronological compilation of a Twitter user’s Tweets) to code for slant.
  - Give less weight to aspects of the Tweet that would require a user to click through.
- Use online bias checkers to help determine slant of news organizations that Tweet, but do not automatically code a Tweet/Twitter user according to the slant of the news sources shared.
- Code Twitter users according to explicitly expressed political affiliations (e.g., independent, conservative) where possible.
- Take into consideration the content of a news source headline within a Tweet, but do not rely solely on the content of linked articles to code for political slant.
- Reference to simply “the government” is insufficient to determine political slant.

***DNA Slant of Tweet and/or Twitter user***

- Tweets that are coded for DNA slant should explicitly address the use of DNA and offer an opinion.
  - Tweets that merely report *that* DNA is being used will be coded as neutral.
- Verb choice when reporting on DNA testing, either in the text of a Tweet or in the headline of a shared article, can and should be used to code for slant; e.g., phrasing such as DNA testing will be done “to help” will generally result in a “For” code.
- Special cases:
  - In general, Tweets that object to migrants *paying* for DNA tests will be coded as “N” for DNA slant.
  - Tweets that are for DNA testing *only* with stringent restrictions are generally coded as A for DNA slant.
  - Tweets that call the use of DNA a “last resort” should be evaluated on a case-by-case basis.

***Zero Tolerance Slant of Tweet and/or Twitter user***

- Association with Republican or Democratic parties alone does not indicate pro- or anti- zero-tolerance stance.
- A pro-family separation stance will be counted as a pro-zero tolerance stance, but an anti-family separation stance cannot be assumed to be an anti-zero tolerance stance.

| **SLANT** | **CODE AND DESCRIPTION** |
| --- | --- |
| Political Slant of Tweet | **Democrat**   - The Tweet expresses support for or affiliation with the Democratic party, its head, or multiple members thereof.   **Republican**   - The Tweet expresses support for or affiliation with the Republican party, its head, or multiple members thereof.   **Anti-Democrat**   - The Tweet expresses disapproval of the Democratic party, its head, or multiple members thereof, without explicitly expressing approval of another party.   **Anti-Republican**   - The Twitter user expresses disapproval of the Republican party, its head, or multiple members thereof, without explicitly expressing approval of another party.   **Neutral**   - (a) Maintains an explicitly neutral stance; or (b) does not address politics |
| Political Slant of Twitter User | **Democrat**   - The Twitter user expresses support for or affiliation with the Democratic party, its head, or multiple members thereof.   **Republican**   - The Twitter user expresses support for or affiliation with the Republican party, its head, or multiple members thereof.   **Anti-Democrat**   - The Twitter user expresses disapproval of the Democratic party, its head, or multiple members thereof, without explicitly expressing approval of another party.   **Anti-Republican**   - The Twitter user expresses disapproval of the Republican party, its head, or multiple members thereof, without explicitly expressing approval of another party.   **Conservative**  **Liberal / Progressive**  **Independent**   - Use if Twitter user explicitly identifies as one of these categories   **Neutral**   - (a) Maintains an explicitly neutral stance; or (b) does not address politics |
| Combined Political Slant of Twitter User and Tweet | **Liberal**   - Both the Twitter user and Tweet are coded as anti-Republican or Democrat (or Twitter user is Liberal/Progressive) - The Twitter user is coded as anti-Republican, Democrat, or Liberal/Progressive and the Tweet is neutral - The Tweet is coded as anti-Republican or Democrat and the Twitter user is neutral - The Twitter user is coded as Independent and the Tweet is coded as anti-Republican or Democrat   **Conservative**   - Both the Twitter user and Tweet are coded as anti- Democrat or Republican (or Twitter user is Conservative) - The Twitter user is coded as anti-Democrat, Republican, or Conservative and the Tweet is neutral - The Tweet is coded as anti-Democrat or Republican and the Twitter user is neutral - The Twitter user is coded as Independent and the Tweet is coded as anti-Democrat or Republican   **Neutral**   - The Twitter user and Tweet are coded as neutral - The Twitter user is coded as Independent and the Tweet is coded as neutral   **Ambiguous**   - The Twitter user is coded as anti-Democrat, Republican, or Conservative and the Tweet is anti-Republican or Democrat. - The Twitter user is coded as anti-Republican, Democrat, or Liberal/Progressive and the Tweet is anti-Democrat or Republican. |
| DNA Slant of Tweet or Twitter User | **For**   - Explicitly supports or has a positive tone towards the use of DNA in immigration contexts.   **Against**   - Explicitly supports or has a positive tone towards the use of DNA in immigration contexts   **Neutral**   - (a) Maintains an explicitly neutral stance; or (b) does not address politics |
| Zero Tolerance Slant | **For**   - Explicitly supports the zero-tolerance policy or family separation or expresses support for stronger restrictions on immigration overall   **Against**   - Explicitly opposes the zero-tolerance policy or expresses support for stronger rights for immigrants   **Neutral**   - (a) Maintains an explicitly neutral stance; or (b) does not address politics |
