# Supplementary material for: Conversations Surrounding the Use of DNA Tests in the Family Reunification of Migrants Separated at the United States-Mexico Border in 2018
Source: Front Genet. 2019 Dec 13;10:1232. doi: 10.3389/fgene.2019.01232 (PMC6927295; doi:10.3389/fgene.2019.01232)
Supplement: Supplementary file 3 [file DataSheet_3.docx]

**CODEBOOK FOR CONTENT**

**OF 27 NEWS SOURCES AND 153 TWEETS**

**COVERING DNA AND FAMILY REUNIFICATION**

**FROM JUNE 1 – JULY 31, 2018**

**CODING PROCESS**

**Two coders separately:**

1. Read or review the main text of the news source or Tweet, including emojis. Note terms or excerpts to define any keywords.
2. Document instances of the content of the news source or Tweet for each topic of interest.

**The two coders reconcile their independent evaluation of the news source or Tweet through discussion.**

**GENERAL RULES**

**Coders will observe the rules for working with Twitter data as already applied to the Codebook for 153 Tweets**

Most codes are Y Yes or N No, unless otherwise marked.

**CONTENT: ETHICS**

| Privacy Concerns | any mention of privacy concerns for the migrants |
| --- | --- |
| Child Consent | any mention of concerns for how to collect informed consent from child migrants |
| Adult Consent | any mention of concerns for how to collect informed consent from adult migrants |
| Rights Violation | any mention of whether the civil or human rights of migrants would be violated or breached through the process of DNA testing |
| Language/Comprehension Barrier | any mention of concerns regarding not being able to communicate the purpose and risks DNA testing due to lack of education, lack of knowledge of DNA, or a language barrier |
| DNA Data Storage/Sharing/Destruction | any mention of concerns regarding if/how DNA specimens will be stored, if/how they will be protected, and if/how they will be destroyed |
| Unexpected Biological Families | any mention of concern that DNA tests could reveal unexpected family relationships |
| Non-Traditional Families | any mention of concern that parents might be adoptive or that guardians might not be biologically related |
| Vulnerable Communities | use of the term “vulnerable” or equivalent term to indicate that the migrant population could be taken advantage of by authorities |
| Uncovering Health Information | any mention of concerns that DNA testing could reveal health information about the migrants |
| Uncertainty of Who is Conducting Tests or Where Tests Are Performed | any mention of concerns regarding either what organization is conducting the DNA testing or where the tests are being performed |
| Parents/Kids Know Each Other Already | any acknowledgment or mention that child migrants might already know their parents or vice versa and hence DNA testing for matching displaced family members would be irrelevant |
| Cultural Beliefs Against DNA | any mention of migrant beliefs or cultural stance regarding DNA testing or use. |

**CONTENT: SCIENCE AND PROCESS**

| What is DNA/Genes | a description or explanation of what DNA or genes are or what they do |
| --- | --- |
| Single Nucleotide Polymorphisms | use of the term SNPs or a description of SNPs |
| Short Tandem Repeats | use of the term STR or a description of STRs |
| Rapid DNA Testing | any mention of the use of Rapid DNA testing or technologies |
| What do Test Results Demonstrate | any comment on what the DNA testing will test for. Labels are as follows:   - P: DNA testing yields results on parentage - K: DNA testing yields results on kinship broader than parent-child relationships - A: DNA testing yields results on ancestry - H: DNA testing yields results on genetic health factors |
| Commercial DNA Test | any potential use of a commercial DNA test or a private entity that might be involved in DNA testing. Labels are as follows:   - L: mention of a specific or general relationship testing DNA analysis laboratory. - R: mention of use of rapid DNA analysis technology company. - A: mention of a specific or general ancestry DNA laboratory. |
| Method of DNA Collection | any mention of some method of DNA collection for testing. Labels are as follows:   - S: saliva and/or spit samples - C: cheek swabs - B: blood tests |
| Who Retrieves Specimen | any comment on what personnel would be interacting with the migrant to retrieve the sample |
| Time in Comparison to Other Reunification Methods | any comparison of time to do DNA testing to other methods of reuniting family (e.g., “shorter than,” “longer time”) |
| Time Frame of Process | specific mention of the amount of time a DNA test might take or time for a certain aspect of the DNA testing process |
| Costs | any mention of the cost of DNA testing or of how expensive/cheap it is relative to other methods of reuniting families. Labels are as follows:   - $$: mention of dollar amount or “spending” - P: use of terms “pay” or “charge” in relation to migrant families - F: use of terms “free,” “reimburse,” or “pro bono” |
| Who Would Pay for Testing | any mention of who will pay for DNA testing or whether services would be donated, volunteered, or provided for free. Labels are as follows:   - C: Company - F: Family - G: Government - N: Non-governmental organization |
| Who would Receive the Sample Report | any mention of who will be receiving a report or information retrieved from the DNA testing |

**CONTENT: LEGAL**

| Prior Use in Other Immigration Programs | any comment of DNA testing being used for immigration purposes prior to the current situation. Labels are as follows:   - P3R: mention of the P3 Refugee program - CAM: mention of the Central American Migrant Minors program - VOL: mention of voluntary submission for immigration to the United States - DET: mention of uploading detainee DNA into CODIS |
| --- | --- |
| Authority and/or Legality of DNA Testing | any mention of whether DNA retrieval is legally allowed or permitted on migrants and/or who has the authority or power or right to administer DNA tests. Labels are as follows:   - A: legality of DNA test for court use - C: question of authority to DNA test a child - I: question of authority to access of DNA information - L: mention of DNA testing per current laws - R: legality/authority (or lack of authority) for DNA testing - J: limiting of DNA testing per judge request |
| Court-Mandated Reunification | any mention of the requirement for DNA tests as mandated by a court order or ruling for reunification |
| HHS or Government DNA Testing | any mention of potential responsibility of HHS or a government entity involved in government DNA testing |
| Storage of DNA Data in Federal Immigration Database | any mention of DNA data used for DNA testing in the context of family separation that might or would be stored in a federal database for immigrants |
| DNA for Future Arrests | any mention of concerns regarding whether DNA data or samples from migrant testing in the context of the migrant family reunifications can be used to arrest or convict migrants or can reveal criminal history about them |
| DNA for Public Safety | any mention of using DNA tests of migrants to promote public safety |
| DNA for Trafficking Detection | any mention of using DNA tests to detect cases of human trafficking |
| DNA to Identity Undocumented Relatives | any mention of using children or the reunification situation to be able to identity or track undocumented relatives in the future |
| Oversight | any mention of the authority overseeing the sensitivity, specificity, or validity of DNA testing |
| External/Legal Advisory for Migrants | any mention of the need for or the use of an external or legal advisor for migrants for DNA testing or reunification |
| Golden State Killer Case | any mention of the Golden State Killer case |
