# Supplementary material for: Conversations Surrounding the Use of DNA Tests in the Family Reunification of Migrants Separated at the United States-Mexico Border in 2018
Source: Front Genet. 2019 Dec 13;10:1232. doi: 10.3389/fgene.2019.01232 (PMC6927295; doi:10.3389/fgene.2019.01232)
Supplement: Supplementary file 4 [file DataSheet_4.docx]

**MEDIA BIAS ASSESSORS**

We selected online bias assessors to evaluate the potential political bias of news sources and content. We are aware of the issues and ongoing critique of facts reported by media and wanted to present the news source data with some external perspective on the subjectivity/objectivity of news sources within our dataset. The following bias assessors are all primarily focused on print news sources, not television or radio. All information in the table below was drawn from the official websites of the listed media bias assessors.

| **Bias Assessor** | **Funding / Project Size** | **Rating Scale and Methodology** | **Sources** |
| --- | --- | --- | --- |
| **AllSides (allsides.com)**  Website founded by John Gable in 2012 | For-profit intentionally funded by a variety of sources to maintain neutrality  Has evaluated nearly 600 sources to date | **Rating Scale:** far left, lean center, center, lean right, or far right  **Methodologies** that may be combined to rate news sources:   - - **Blind bias survey:** Readers across the political spectrum are recruited to read and rate multiple articles from a single, undisclosed source. Ratings are normalized to reflect the distribution of political slant across the nation, and the average rating of several articles results in the rating of the source.   - **Editorial review:** Reviews of articles conducted by staff from across the political spectrum, sometimes done blind.   - **Third-party data:** Third-party sources AllSides deems credible.   - **Independent research:** Online research conducted by the AllSides team.   - **Community feedback**: Site users report their level of (dis)agreement with ratings; discrepancies between community feedback and bias ratings prompt AllSides to reevaluate sources. | Information was drawn from the following website sections: “What is AllSides?,” “The Dark Ages of the Internet,” “How AllSides Rates Media Bias: Our Methods,” “FAQs,” “Media Bias Ratings,” “Our Team”  See: AllSides, 2019. https://www.allsides.com/unbiased-balanced-news. |
| **Media Bias/Fact Check, LLC (MBFC News)**  Independent online media outlet founded by Dave van Zandt in 2015 | Funded by advertising and donations; staffed by volunteers  Has evaluated over 2,500 sources to date | - - **Rating scale: Left bias, left-center bias, least biased, right-center bias, right bias.** Articles are rated 0 (least biased) to 10 (most biased) in four categories, *biased wording/headlines*, *factual/sourcing*, *story choices*, and *political affiliation*   - The average of these scores together with the direction of the bias makes up the bias score.   **Methodology:**   - - Ratings of sources are based on a minimum of 10 headlines and 5 sources.   - Bias assessors start with news articles and move on to editorial and opinion pieces, reviewing content until the bias is clear.   - Reader votes on bias are collected and displayed; large discrepancies between reader votes and Media Bias / FactCheck’s rating prompt re-evaluation of sources. | Information was drawn from the following website sections: “About,” “Methodology,” “FAQs”  See: Media Bias/Fact Check. Accessed February 12, 2018. https://mediabiasfactcheck.com. |
| **Ad Fontes Media, Inc.**  Public Benefit Corporation (PBC) founded by Vanessa Otero, JD in 2018 | Funded by sale of licenses and prints as well as donations; staff consists of founder Vanessa Ortero, JD with occasional help from volunteers.  Has evaluated 104 sources to date | **Rating scale:**   - - **Bias:** the horizontal axis of the Media Bias Chart is rated from “most extreme left” to “most extreme right.”   - **Quality:** the vertical axis of the Media Bias Chart is rated on a numerical scale from 0 to 64 with 0 corresponding to “contains inaccurate / fabricated info” and 64 corresponding to “original fact reporting”   **Methodology:**   - - At least 10 articles are rated per source.   - **Bias** is rated on the basis of: Bias ratings for topic selection, topic presentation, overall article bias as compared to other articles on the same topic, and number of instances of biased political position, characterization, and terminology in each sentence.   - **Quality** is rated on the basis of: A score of 1 (inaccurate/fabricated info) through 8 (original fact reporting) for all article elements (e.g., title), a score of 1 (true) through 5 (false) for veracity and a score of 1 (fact statement) through 5 (opinion statement) for expression for each sentence in the article, and a count of “unfairness instances” (inclusion of content that, regardless of presentation and factuality, contributes to bias in the context of the topic being covered). The combined score provides the final Quality rating between 0 to 64. | Information was drawn from the following website sections: “Ad Fontes Media,” “How Ad Fontes Ranks Media Sources,” “The Chart, Version 3.0: What, Exactly, Are We Reading?”, “Media Bias Chart 4.0”  See: Otero, Vanessa. ad fontes media, 2019. https://www.adfontesmedia.com. |
