# Supplementary material for: Conversations Surrounding the Use of DNA Tests in the Family Reunification of Migrants Separated at the United States-Mexico Border in 2018
Source: Front Genet. 2019 Dec 13;10:1232. doi: 10.3389/fgene.2019.01232 (PMC6927295; doi:10.3389/fgene.2019.01232)
Supplement: Supplementary file 5 [file Presentation_1.pdf]

SUPPLEMENTAL TABLES

Table S1. News article search sources

**Search(es) leading to included articles**	**Total number of articles (*N*=526)**	**Number of unique articles (*N*=263)**	**Number relevant articles (*N*=237)**
Nexis Uni	362	129	108
ProQuest + Nexis Uni	1	1	1
ProQuest	26	23	16
ProQuest + Westlaw Next	10	9	9
Westlaw Next	99	76	50
Nexis Uni + Westlaw Next	24	22	16
ProQuest + Nexis Uni + Westlaw Next	4	3	3
Ad hoc articles			34

Table S2. Eligibility of news articles for analysis

**Search(es) leading to included articles**	**TV news**	**Press releases**	**Article by authors**	**Articles without online access**	**Accessible articles for analysis (*N*=183)**
Nexis Uni	29	18	--	2	59
ProQuest + Nexis Uni	--	--	--	--	1
ProQuest	--	--	--	--	16
ProQuest + Westlaw Next	--	--	--	--	9
Westlaw Next	--	--	1	1	48
Nexis Uni + Westlaw Next	--	--	--	--	16
ProQuest + Nexis Uni + Westlaw Next	--	--	--	--	3
Ad hoc articles	--	3	--	--	31

Table S3. Bias in media sources

**Bias according to allsides.com or mediabiasfactcheck.com**	**Number of articles mentioning use of DNA (*N*=183)**	**Number of articles covering DNA testing with context (*N*=70)**	**Number of articles covering DNA testing addressing science, process, legality, or ethics (*N*=27)**
Left-leaning sources	96	42	17
Right-leaning sources	30	8	2
Least biased sources	24	9	6
Sources not evaluated by bias assessors	33	11	2

Table S4. Depth of context on DNA testing in articles

**Search(es) leading to included articles**	**Lack of DNA context**	**Sufficient mention of DNA testing for analysis (*N*=70)**	**Discussion of science or legal authority for DNA testing in article (*N*=27)**
Nexis Uni	43	16	6
ProQuest + Nexis Uni	--	1	--
ProQuest	13	3	--
ProQuest + Westlaw Next	6	3	1
Westlaw Next	37	11	1
Nexis Uni + Westlaw Next	10	6	4
ProQuest + Nexis Uni + Westlaw Next	1	2	--
Ad hoc articles	3	28	15

Table S5. Characteristics of news articles analyzed

**Search(es) leading to included articles**	**Articles analyzed (*N*=70)**	**Word count range**	**Date range**
Nexis Uni	16	294-1,417	June 23 – July 16
ProQuest + Nexis Uni	1	1,346	July 6
ProQuest	3	844-1,909	July 7 – July 23
ProQuest + Westlaw Next	3	681-1,325	July 2 – July 19
Westlaw Next	11	138-1,507	June 22 – July 28
Nexis Uni + Westlaw	6	652-1,352	June 21 – July 10
ProQuest + Nexis Uni + Westlaw	2	971-1,134	July 12 – July 14
Ad hoc articles	28	289-1,995	June 21 – July 17

Table S6. Zero-tolerance slant of articles

**Search(es) leading to included articles**	**Articles analyzed (*N*=70)**	**Pro- zero-tolerance (*N*=5)**	**Anti- zero-tolerance (*N*=32)**	**Neutral (*N*=33)**
Nexis Uni	16	2	5	9
ProQuest + Nexis Uni	1	--	1	--
ProQuest	3	--	3	--
ProQuest + Westlaw Next	3	--	3	--
Westlaw Next	11	3	3	5
Nexis Uni + Westlaw Next	6	--	2	4
ProQuest + Nexis Uni + Westlaw Next	2	--	--	2
Ad hoc articles	28	--	15	13

Table S7. Slant on DNA testing of articles

Search(es) leading to included articles	Articles analyzed (*N*=70)	Pro- DNA testing (*N*=10)	Anti- DNA testing (*N*=21)	Neutral (*N*=39)
Nexis Uni	16	2	3	11
ProQuest + Nexis Uni	1	--	1	--
ProQuest	3	1	1	1
ProQuest + Westlaw Next	3	1	1	1
Westlaw Next	11	4	1	6
Nexis Uni + Westlaw Next	6	1	3	2
ProQuest + Nexis Uni + Westlaw Next	2	--	--	2
Ad hoc articles	28	1	11	16

Table S8. Experts consulted in news articles

Expert	Number of articles
Academic	15
Forensic	2
Government	12
Industry	17
Law enforcement	--
Non-governmental organization	15
No expert consulted	48

Table S9. Topics covered regarding DNA testing in news articles

DNA testing application topic	Number of articles
Collection of detainee DNA for CODIS	4
Rapid DNA testing of families at border prior to separation	6
DNA testing of families for reunification following separation (rapid or traditional)	25
Ancestry DNA testing	5
No specific coverage	45

Table S10. Excerpts of coverage of DNA and identity testing in the news articles

**News source (aspect of DNA covered)**	**Quotation**	**Citation**
AH03: June 22: Family DNA testing at the border would be an ethical quagmire (STRs)	"The tests commonly used to determine paternity or maternity rely on a different method that provides a simple yes or no answer. Those comparisons are based on non-coding portions of the genome, the same kind of arrays found in criminal databases."	Molteni M, *Wired*
AH06: June 25: Could DNA testing reunite immigrant families? Get the facts (DNA, SNPs)	"Your genetic fingerprint is a blend of DNA from your biological mom and dad. That means every gene in your body comes from one of your parents. To help migrant families, parents and children would all provide samples of their genetic material. For 23andMe tests, that would require spitting in a tube; MyHeritage kits use a cheek swab. The companies then examine roughly 700,000 of the three billion basic units of genetic material that make up the human genome, explains Miguel Vilar, lead scientist for National Geographic's Genographic Project."	Wei-Haas M, *National Geographic*
AH08: June 26: Genetic testing to reunite immigrant families raises issues of privacy and consent (DNA, SNPs, STRs)	"Paternity/maternity tests look at spots in the genome with so-called short-tandem repeats. These are areas where sequences of nucleotides—the A, C, G and T letters that make up DNA—are repeated potentially tens of times, varying in length from family to family, Torkamani says. There's a lot of variability among family groups but also a lot of consistency between parent and child, he notes. A relationship can be established by looking at only a few dozen genome locations."	Weintraub K, *Scientific American*
AH14: July 2: DNA testing is not the way to reunite families (STRs)	"If any DNA testing is required (for example for young infants), it should be restricted to the minimum number of markers necessary to inform the relationship with the parent."	Hamosh A, et al. *Baltimore Sun*
NU12: July 6: Which Company Will Test the DNA of Separated Families? (STRs)	"The DNA test HHS is using looks at 20 or so locations in the genome. Judging from a description of the tests, these locations are likely STRs, or 'short tandem repeats,' where stretches of DNA are naturally repeated. STRs are useful for establishing parent or sibling relationships but are not as useful for grandparents, aunts, uncles, or more distant relations."	Zhang S, *Atlantic Online*
AH29: July 7: U.S. using DNA testing in effort to reunite families it separated (STRs)	"Such testing looks at a small portion of DNA, known as 'short tandem repeats,' to identify inherited regions of DNA that can vary from person to person."	Beasley D, Cooke K. *Reuters*
NU19: July 9: DNA tests used to reunite migrant families spark worries they'll be used for much more (STRs)	"The most likely type of test would be a Short Tandem Repeat analysis or STR, the same type of testing the FBI does in criminal cases."	Weise E, Suppe R, Gomez A, *USA Today*
AH30: July 10: DNA collection is not the answer to reuniting families split apart by Trump's "zero tolerance" program (DNA)	"Our DNA contains our entire genetic makeup. It can reveal where our ancestors came from, who we are related to, our physical characteristics, and whether we are likely to get various genetically-determined diseases. Researchers have also theorized DNA may predict race, intelligence, criminality, sexual orientation, and even political ideology."	Lynch J, *Electronic Frontier Foundation*

AH32: July 11: DNA testing on separated migrant families needs robust privacy safeguards (STRs)	“Typically, CODIS’s DNA profiles are drawn from 20 highly variable locations on the human chromosomes, which can provide enough information to determine a match or close relative. These profiles consist of data drawn from noncoding DNA, meaning that the locations of the DNA sequenced don’t code for proteins. (Consumer genetic profiles, by contrast, typically rely on coding DNA to determine an individual’s traits and health risks).”	Ram N, *Slate*

Table S11. Excerpts of coverage of legal authority for DNA testing in the news articles

News source	Quotation	Citation
AH03: June 22: Family DNA testing at the border would be an ethical quagmire	"Current law gives people legally seeking US citizenship the option to order DNA tests as part of their visa application if other forms of documentation are missing or their veracity is in doubt."	Molteni M, *Wired*
AH04: June 22: DNA testing could reunite families at the border-and fuel surveillance	"Immigrants to the US will often submit DNA testing to prove familial relationships, but that testing is strictly voluntary. While the United States Citizenship and Immigration Services has lobbied for the power to force genetic testing in cases of suspected fraud, it still can't compel immigrants who don't want to be tested. If DNA testing becomes a more routine part of the immigration system, family reunification could be used as a way around that restriction."	Brandom R, Becker R, *The Verge*
WL03: June 27: Effort to use DNA tests to reunite migrant families is paused	"Children under age 18 usually need parental permission to submit their DNA for a test, but since detained children become a ward of the state, the federal government would have to act as a temporary guardian to grant consent."	Ho C, *San Francisco Chronicle*
AH12: June 29: A lesser-known DNA test that can help reunite immigrant parents with detained children	"The Office of Refugee Resettlement told Congress in April that of the 7,000 children returned to relatives in fall 2017, it had lost track of 1,475 children. A PBS Frontline investigation called 'Trafficked in America' that aired in April also discovered cases of teenagers being released to labor traffickers by the ORR. DNA testing kits could solve the issue by identifying relatives accurately."	Song K, *CNBC*
AH23: July 5: Reported DNA testing on migrants raises questions, concerns	"The US government has used DNA testing in immigration matters since the 1990s, and its legal authority to use DNA information in this regard has only grown in recent years. Since 2009, the government has had broad authority to collect DNA samples from arrestees and importantly, from non-US persons who are detained, even if they're not arrested."	Ray T, *GenomeWeb*
NU12: July 6: Which Company Will Test the DNA of Separated Families?	"HHS officials say they are resorting to DNA to meet the court-mandated deadline of reuniting children younger than 5 by Tuesday, as verifying relationships through documents like birth certificates would take too long."	Zhang S, *Atlantic Online*
PQ18: July 8: U.S. seeks more time to reunite families	"Authorities say they are conducting DNA testing and background checks to protect children as required by a bipartisan anti-trafficking law enacted in 2008."	Sacchetti M, *The Washington Post*
NU19: July 9: DNA tests used to reunite migrant families spark worries they'll be used for much more	"The U.S. Department of Citizenship and Immigration began discussing the use of DNA testing in 2000 to aid in family reunification cases, but there was a lack of clarity around whether it would be legal."	Weise E, Suppe R, Gomez A, *USA Today*
AH30: July 10: DNA collection is not the answer to reuniting families split apart by Trump's "zero tolerance" program	"DHS still doesn't appear to have legal authority to collect DNA samples from anyone younger than 14. Children younger than 14 should not be deemed to have consented to DNA collection. And under these circumstances, parents cannot consent to the collection of DNA from their children because the federal government has admitted it has already lost track of which children are related to which adults."	Lynch J, *Electronic Frontier Foundation*

AH31: July 10: Government told immigrant parents to pay for DNA tests to get kids back, advocate says	"The tests are being administered by a private contractor on behalf of the Department of Health and Human Services' Office of Refugee Resettlement, which oversees the care and housing of children."	Glawe J, Rawnsley A, *The Daily Beast*
NU23: July 10: The latest: US judge calls for shortening wait for families	"The judge said the government can use DNA testing but solely when necessary and with parental consent. The sampling should be destroyed in 7 days and not used for any other purpose."	--, *Associated Press*
AH32: July 11: DNA testing on separated migrant families needs robust privacy safeguards	"The national missing persons program provides some legal precedent for the collection and use of genetic data for the limited purpose of reunifying families."	Ram N, *Slate*
AH34: June 25: Why there's a deep cultural aversion to DNA testing, even when it can reunite separated immigrant families	"The government has argued that DNA testing is part of an extensive vetting process to protect children."	Richards SE, *Time*

Table S12. Excerpts of coverage of oversight for DNA testing in the news articles

News source	Quotation	Citation
NU02: June 21: 23andMe donating DNA kits to help reunite migrant families	"23andMe uses at-home saliva samples sent to accredited labs to determine ancestry estimates 'down to the 0.1%,' according to the company's website."	Sanchez T, *Mercury News*
AH12: June 29: A lesser-known DNA test that can help reunite immigrant parents with detained children	"There are problems giving it to a commercial testing company. They do recreational genetics."	Song K, *CNBC*
AH23: July 5: Reported DNA testing on migrants raises questions, concerns	"The FBI normally does not permit samples into CODIS that haven't followed [Scientific Working Group on DNA Analysis Methods] and [American Society of Crime Laboratory Directors] guidelines."	Ray T, *GenomeWeb*
AH30: July 10: DNA collection is not the answer to reuniting families split apart by Trump's "zero tolerance" program	"Are they collecting a sample via a swab of the cheek? Is collection coerced or is it with the consent and assistance of the undocumented person? Once the sample is collected, how is it processed? Is it processed in a certified lab? Is it processed using a Rapid DNA machine? How is chain of custody tracked, and how is the collecting entity ensuring samples aren't getting mixed up?"	Lynch J, *Electronic Frontier Foundation*

Table S13. Mention of prior use of DNA in the news articles

News source	Prior use mention	Political slant of article (mediabiasfactcheck.com)	Slant on DNA testing	Citation
AH03: June 22: Family DNA testing at the border would be an ethical quagmire	Central American Migrant Minors program DNA requirement<br><br>Mandated detainee DNA collection for CODIS	Center-left	Neutral	Molteni M, *Wired*
AH04: June 22: DNA testing could reunite families at the border—and fuel surveillance	Voluntary provision of DNA for support of immigrant visa petition	Center-left	Against	Brandom R, Becker R, *The Verge*
NU05: June 27: Immigrant advocates turn down DNA tests	DNA-ProKids	Least bias	Against	*Voice of America*
AH23: July 5: Reported DNA testing on migrants raises questions, concerns	Mandated detainee DNA collection for CODIS<br><br>Voluntary provision of DNA for support of immigrant visa petition	Not listed	Neutral	Ray T, *GenomeWeb*
NU08: July 5: DNA tests for separated families slammed by immigration advocates	Voluntary provision of DNA for support of immigrant visa petition	Center-left	Against	*NBC News*
NU12: July 6: Which company will test the DNA of separated families?	Central American Migrant Minors program DNA requirement<br><br>Priority 3 family reunification program DNA requirement	Center-left	Against	Zhang S, *Atlantic Online*
AH29: July 7: U.S. using DNA testing in effort to reunite families it separated (STRs)	Unaccompanied alien children DNA testing<br><br>Voluntary provision of DNA for support of immigrant visa petition	Least bias	Neutral	Beasley D, Cooke K. *Reuters*
NU19: July 9: DNA tests used to reunite migrant families spark worries they'll be used for much more	Mandated detainee DNA collection for CODIS	Center-left	Against	Weise E, Suppe R, Gomez A, *USA Today*
AH31: July 10: Government told immigrant parents to pay for DNA tests to get kids back, advocate says	Central American Migrant Minors program DNA requirement	Left	Neutral	Glawe J, Rawnsley A, *The Daily Beast*

	Priority 3 family reunification program DNA requirement<br><br>Unaccompanied alien children DNA testing	

Table S14. Coverage of DNA testing process, ethics and legal authority in news articles

**News article focus**	**Number of articles (*N*=27)**	**Number of articles mentioning method of DNA collection (*N*=19)**	**Number of articles mentioning who pays for DNA test (*N*=10)**	**Number of articles mentioning ethics topics (*N*=134)**	**Number of articles mentioning legality topics (*N*=71)**
Commercial DNA test offer	13	8 (61.5%)	9 (69.2%)	67 (5.3 per article)	26 (2 per article)
HHS announcement of DNA testing	4	3 (75.0%)	--	21 (5.3 per article)	13 (3.3 per article)
Both	7	7 (100%)	1 (14.3%)	37 (5.3 per article)	25 (3.5 per article)
Neither	3	1 (33.3%)	--	9 (3 per article)	7 (2.3 per article)

Table S15. Twitter search strings

Search	Number Tweets in search results (*N*=219)	Duplicate Tweets	Relevant Tweets (*N*=153)
"DNA" + any of 17 trending hashtags	55	1	40
"DNA" + any of "migrant" "immigrant" "immigration" "refugee" "illegals"	160	1	109
Both searches	4	--	4

Table S16. Characteristics of Twitter search results

Search	Tweets analyzed (*N*=153)	Original Tweets (*N*=133)	Re-Tweets (*N*=20)	Including direct mentions (*N*=26)	Including linked news articles (*N*=84)	Use of images (other than linked articles) (*N*=15)	Use of emojis in text (*N*=10)
"DNA" + any of 17 trending hashtags[a]	40	32	8	11	15	6	7
"DNA" + any of "migrant" "immigrant" "immigration" "refugee" "illegals"	109	99	10	14	67	9	3
Both searches	4	2	2	1	2	--	--

[a] #keepfamiliestogether; #familiesbelongtogether; #reunitefamilies; #reunitethefamilies; #reunitefamiliesnow; #reunification; #separationoffamilies; #childrenincages; #familyseparation; #reuniteeverychild; #returnthechildren; #bordercrisis; #childrensconcentrationcamps; #childtrafficking; #humantrafficking; #illegalimmigration; #buildthewallnow

Table S17. Use of social media hashtags in Tweets

**Hashtag theme**	**Hashtag(s) (* denotes hashtags used in original search)**	**Number of Tweets**	**Number of re-Tweets**
General reference to immigration	#asylumseekers<br>#immigrant<br>#immigrantchildren<br>#immigration<br>#USMexicoborder	7	135
General reference to border control	#buildthewall<br>#istandwithice<br>#illegalimmigration*	4	364
Reference to human trafficking	#childtrafficking*<br>#humantrafficking*<br>#endtrafficking<br>#sextrafficking<br>#traffickstop	11	771
Specific to family separation	#childrenincages*<br>#familyseparation*<br>#babyjails<br>#children<br>#helpsavethechildren<br>#savethechildren<br>#singlestagingareanow	11	733
Specific to family reunification	#familiesbelongtogether*<br>#keepfamiliestogether*<br>#reuniteeverychild*<br>#reunitefamilies*<br>#reunitefamiliesnow*<br>#reunitethefamilies*<br>#familiesbelongstogether<br>#reunitethe2300	37	4,558
General reference to elections	#vote<br>#winning	2	49
Support of conservative candidates	#patriotsfight<br>#rednationrising	2	92
Support of democratic candidates	#bluewave<br>#democrats	2	175
Conspiracy movements	#QAnon<br>#WWG1WGA<br>#pedogate	5	301
Support of anti-conservative agenda	#resist<br>#theresistance<br>#fightlikeamother<br>#wevegotquestions	3	100
Directed to President Donald J Trump or his campaign	#Trump<br>#@POTUS<br>#potus<br>#thanksdonald<br>#trumpcrimesagainstchildren<br>#trumptrain<br>#maga<br>#traitortot	10	572
Support of law enforcement	#backtheblue	1	--

Intended to evoke emotion or for affect	#evil<br>#monsters<br>#psychologicaltrauma<br>#unintendedconsequences<br>#vermin	3	115
News update	#breaking<br>#update<br>#thursdaythoughts	3	263
Science	#DNA	4	29
Other specific organizations and individuals	#stzork<br>#maddow<br>#unitednations	3	219

Table S18. Use of direct mentions in Tweets

**Hashtag theme**	**Direct mention(s)**	**Number of Tweets**	**Number of re-Tweets**
Commercial DNA companies	@23andMe<br>@23andMeresearch<br>@FamilyTreeDNA<br>@MyHeritage	2	15
Government	@CBP<br>@DHS<br>@DHSgov<br>@ICEgov<br>@SecAzar<br>@SecNielsen	5	262
President of the United States and staff	@realDonaldTrump<br>@POTUS<br>@DonaldJTrumpJr<br>@SebGorka	8	2,055
Politician	@HillaryClinton	1	49
Non-governmental organization	@ACLU<br>@RAICESTEXAS	3	768
Journalists	@cnbctech<br>@CNNpolitics<br>@dailycaller<br>@justinglawe<br>@stassaedwards<br>@thedailybeast<br>@westjournalism	8	998

Table S19. Extrapolated purpose of Tweets

Purpose	Number of Tweets (*N*=153)	Number of re-Tweets (*N*=31,976)
Advocates	23 (15.0%)	2,388 (7.5%)
Announces	1 (0.7%)	458 (1.4%)
Calls upon	12 (7.8%)	4,937 (15.4%)
Comments	76 (49.7%)	23,296 (72.9%)
Expresses	16 (10.5%)	2,265 (7.1%)
Hints	4 (2.6%)	3,361 (10.5%)
Questions	9 (5.9%)	436 (1.4%)
Reports	87 (56.9%)	24,798 (77.6%)
Shares	81 (51.6%)	11,091 (34.7%)
Solicits	1 (0.7%)	582 (1.8%)
Suggests	8 (5.2%)	55 (0.2%)

Table S20. Slant on zero-tolerance policy in Twitter

Political slant	Number of Tweets	Pro- zero-tolerance policy	Anti- zero-tolerance policy	Neutral
Conservative	52	46	--	6
Liberal / Progressive	80	--	69	11
Ambiguous	1	--	1	--
Neutral in Both	20	2	5	13
Total	153	48 (31.4%)	75 (49.0%)	30 (19.6%)
Total re-Tweets	*31,976*	*12,093 (37.8%)*	*15,293 (47.8%)*	*4,590 (14.4%)*

Table S21. Slant on DNA testing in Twitter

Political Slant	Number of Tweets	Pro- DNA testing	Anti- DNA testing	Neutral
Conservative	52	42	--	10
Liberal / Progressive	80	21	24	35
Ambiguous	1	1	--	--
Neutral in both	20	7	3	10
Total Tweets	153	71 (46.4%)	27 (17.6%)	55 (35.9%)
Total re-Tweets	*31,976*	*16,727 (52.3%)*	*5,435 (17.0%)*	*9,815 (30.7%)*

Table S22. Topics and concepts covered in Tweets

People	Organizations	Aspects of family separation process and documentation	Immigration topics and terms	Social topics
Donald J Trump (31)	Genomics companies (20)	Plan (40)	"Migrant" (40)	Biological family (45)
Jeff Sessions (10)	Department of Health and Human Services (7)	Cost (38)	Immigration fraud (35)	Ethics (18)
Dana Sabraw (7)	Immigration and Customs Enforcement (7)	Timeline (10)	Human trafficking (33)	Consent (5)
Elizabeth Warren (6)	American Civil Liberties Union (4)	Court order (8)	Immigration policy (26)	Privacy (4)
Barack Obama (5)	Department of Homeland Security (3)	Database (6)	"Illegal" (17)	Surveillance (2)
QANON (5)	Government contractor (3)	Deadline (6)	Asylum (6)	Deterrence (1)
Alex M Azar (3)	Customs and Border Protection / Border patrol (2)	Record-keeping (5)	"Refugee" (4)	
Hillary Clinton (2)	Immigration and Naturalization Service / U.S. Citizenship and Immigration Services (2)	Biometric data (3)	Sponsorship (1)	
Kirstjen Nielsen (2)	Office of Refugee Resettlement (2)			
George W Bush (1)	Refugee and Immigrant Center for Education and Legal Services (2)			
Rudy Giuliani (1)	Center for Immigration Studies (1)			
Beto O'Rourke (1)	Family Research Council (1)			
Jackie Speier (1)	United Nations (1)			
Immigration attorney (1)				

Table S23. Coverage of costs and financial responsibilities for DNA testing in Tweets

Topic	Number of Tweets (*N*=153)	Political slant	Zero-tolerance policy slant	DNA testing slant
Costs (mention of $ amounts), "spending"	12 (7.8%)	10 liberal<br>1 conservative<br>1 neutral	10 against<br>1 for<br>1 neutral	3 against<br>3 for<br>5 neutral
Family burden ("pay," "charging")	26 (17.0%)	22 liberal<br>4 neutral	22 against<br>4 neutral	3 against<br>1 for<br>22 neutral
Offer of "free" services, "pro bono," or reimbursement	6 (3.9%)	5 liberal<br>1 neutral	5 against<br>1 neutral	1 against<br>3 for<br>2 neutral
Government should pay	2 (1.3%)	2 liberal	2 against	1 against<br>1 for
NGO should pay	1 (0.7%)	liberal	against	For
Company could pay	12 (7.8%)	10 liberal<br>2 neutral	9 against<br>3 neutral	1 against<br>9 for<br>2 neutral
Family has to pay	30 (19.6%)	25 liberal<br>5 neutral	25 against<br>5 neutral	3 against<br>1 for<br>24 neutral

Table S24. Coverage of secondary uses of DNA in Tweets

Topic	Number of Tweets (*N*=153)	Political slant	Zero-tolerance policy slant	DNA testing slant
DNA for future arrests	2 (1.3%)	2 liberal	1 against<br>1 neutral	2 against
DNA for detecting wanted criminals	2 (1.3%)	2 conservative	2 for	1 for<br>1 neutral
DNA for detecting human trafficking	37 (24.2%)	1 liberal<br>33 conservative<br>3 neutral	2 against<br>31 for<br>4 neutral	1 against<br>32 for<br>4 neutral
DNA for detecting undocumented relatives	--	--	--	--
